# Author Correction: Temporal dynamic reorganization of 3D chromatin architecture in hormone-induced breast cancer and endocrine resistance

**DOI:** 10.1038/s41467-020-15789-6

**Published:** 2020-04-20

**Authors:** Yufan Zhou, Diana L. Gerrard, Junbai Wang, Tian Li, Yini Yang, Andrew J. Fritz, Mahitha Rajendran, Xiaoyong Fu, Gary Stein, Rachel Schiff, Shili Lin, Seth Frietze, Victor X. Jin

**Affiliations:** 1 Department of Molecular Medicine, University of Texas Health San Antonio, San Antonio, TX 78229 USA; 2 MLRS Department, University of Vermont, Burlington, VT 05405 USA; 3 Department of Pathology, Oslo University Hospital—Norwegian Radium Hospital, 0310 Montebello, Oslo Norway; 4 Department of Biochemistry, University of Vermont, Burlington, VT 05405 USA; 5 Department of Molecular and Cellular Biology, Baylor College of Medicine, Houston, TX 77030 USA; 6 Department of Medicine, Baylor College of Medicine, Houston, TX 77030 USA; 7 Lester and Sue Smith Breast Center, Baylor College of Medicine, Houston, TX 77030 USA; 8 Dan L. Duncan Comprehensive Cancer Center, Baylor College of Medicine, Houston, TX 77030 USA; 9 Department of Surgery, University of Vermont Larner College of Medicine, 89 Beaumont Avenue, Given C401, Burlington, Vermont 05405 USA; 10 Department of Statistics, The Ohio State University, Columbus, OH 43210 USA

**Keywords:** Cancer genomics, Epigenomics

Correction to: *Nature Communications* 10.1038/s41467-019-09320-9, published online 03 April 2019.

The original version of this Article omitted from the author list the 9th author ‘Gary Stein’, who is from the ‘Department of Biochemistry, University of Vermont Larner College of Medicine’. Consequently, the following was added to the Author Contributions: ‘Y.Z. performed the data analysis. V.X.J., Y.Z. and S.F. wrote the manuscript, with all authors including J.W., M.R., X.F., G.S., R.S. and S.L. contributing to writing and providing the feedback.' This has been corrected in both the PDF and HTML versions of the Article.

