# Supplementary material for: Temporal dynamic reorganization of 3D chromatin architecture in hormone-induced breast cancer and endocrine resistance
Source: Nat Commun. 2019 Apr 3;10:1522. doi: 10.1038/s41467-019-09320-9 (PMC6447566; doi:10.1038/s41467-019-09320-9)
Supplement: Supplementary file 1 — Supplementary Information [file 41467_2019_9320_MOESM1_ESM.pdf]

Supplementary Information

Temporal dynamic reorganization of 3D chromatin architecture in hormone-induced breast cancer and endocrine resistance

Zhou et al.

Supplementary Figure 1. Negative Binomial dispersion of T0/T1 two replicates

After TCC raw data were mapped to human hg19 genome, read pairs of two replicates of T0 and T1 were treated with diffHiC[1] to estimate the Negative Binomial dispersion for the modelling biological variability. In the figure, CPM means Count Per Million. The variation is obviously decreased when the counts are increased due to more precise.

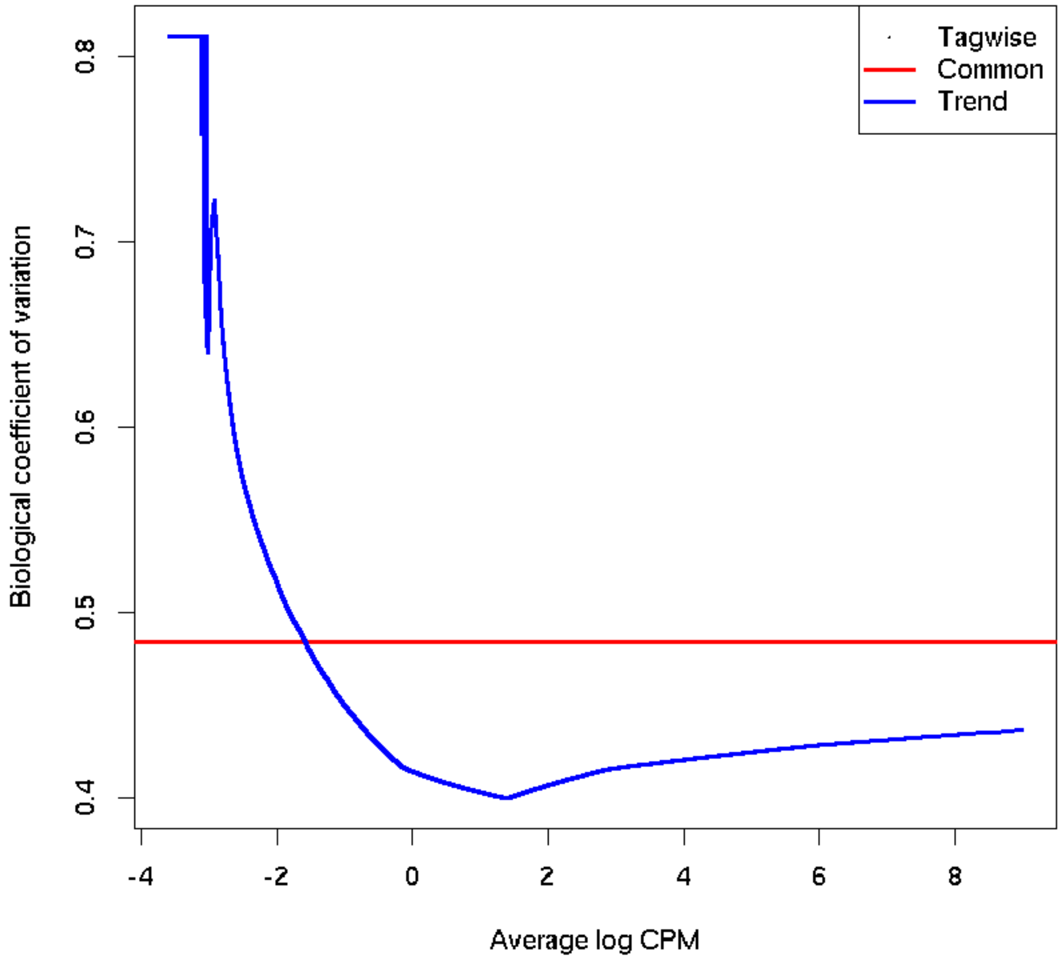

Supplementary Figure 2. Compartment matrix of T0 by chromosome, chr2-13

Compartment matrix and first eigenvectors of T0, chrosome 2 to chrosome 13

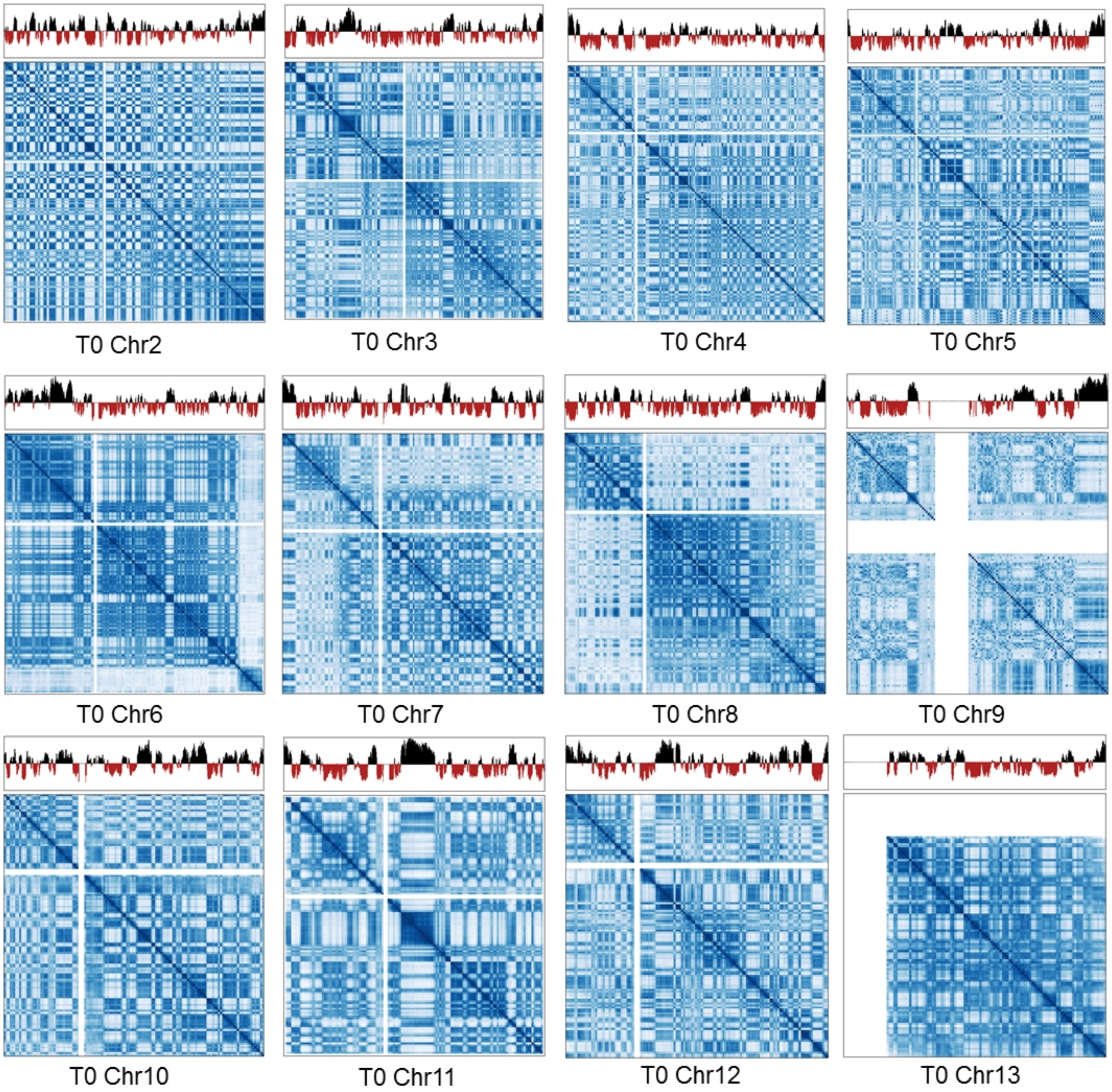

Supplementary Figure 3. Compartment matrix of T0 by chromosome, chr14-X

Compartment matrix and first eigenvectors of T0, chrosome 14 to chrosome X

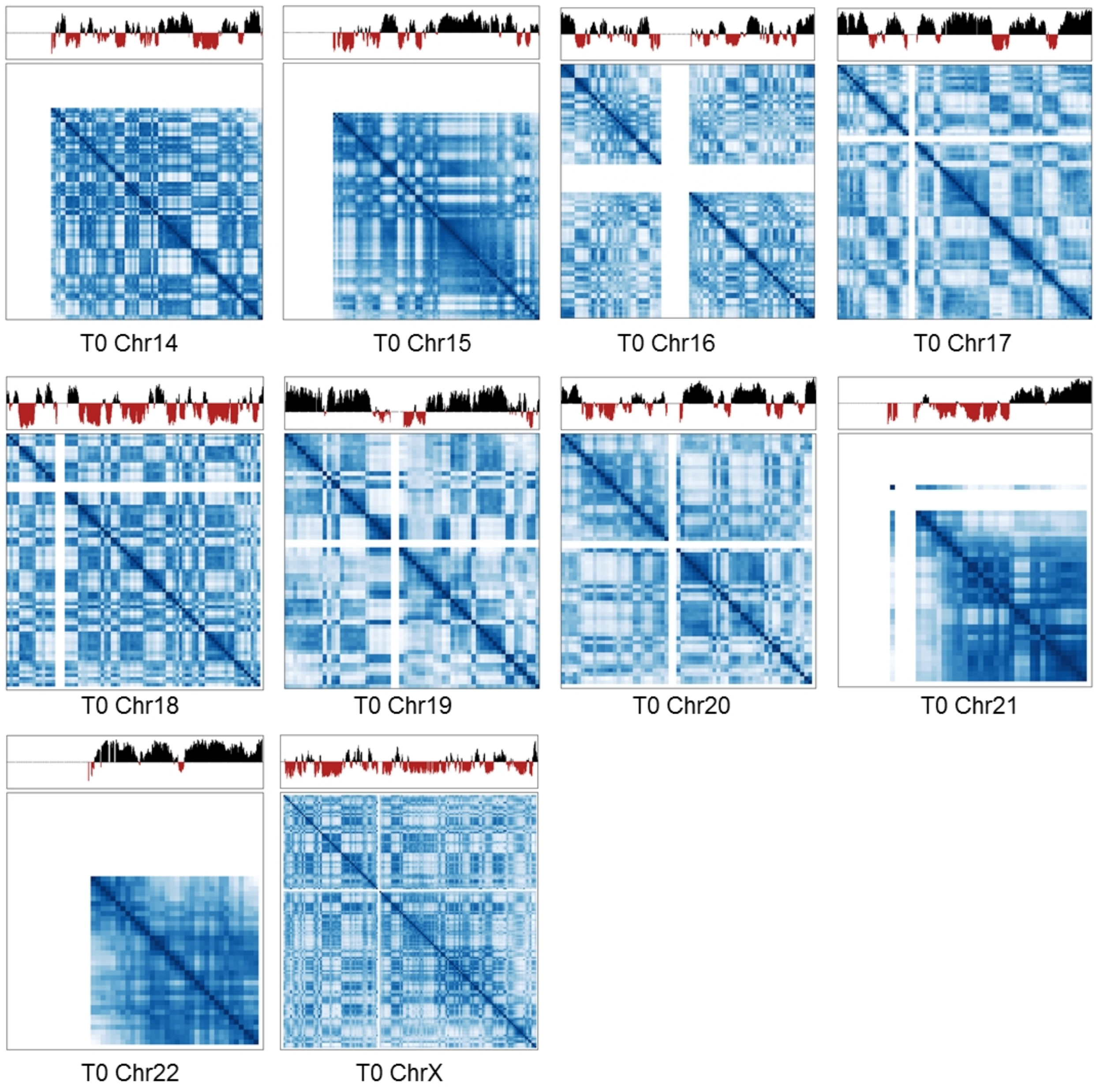

Supplementary Figure 4. Compartment matrix of T1 by chromosome, chr2-13

Compartment matrix and first eigenvectors of T1, chrosome 2 to chrosome 13

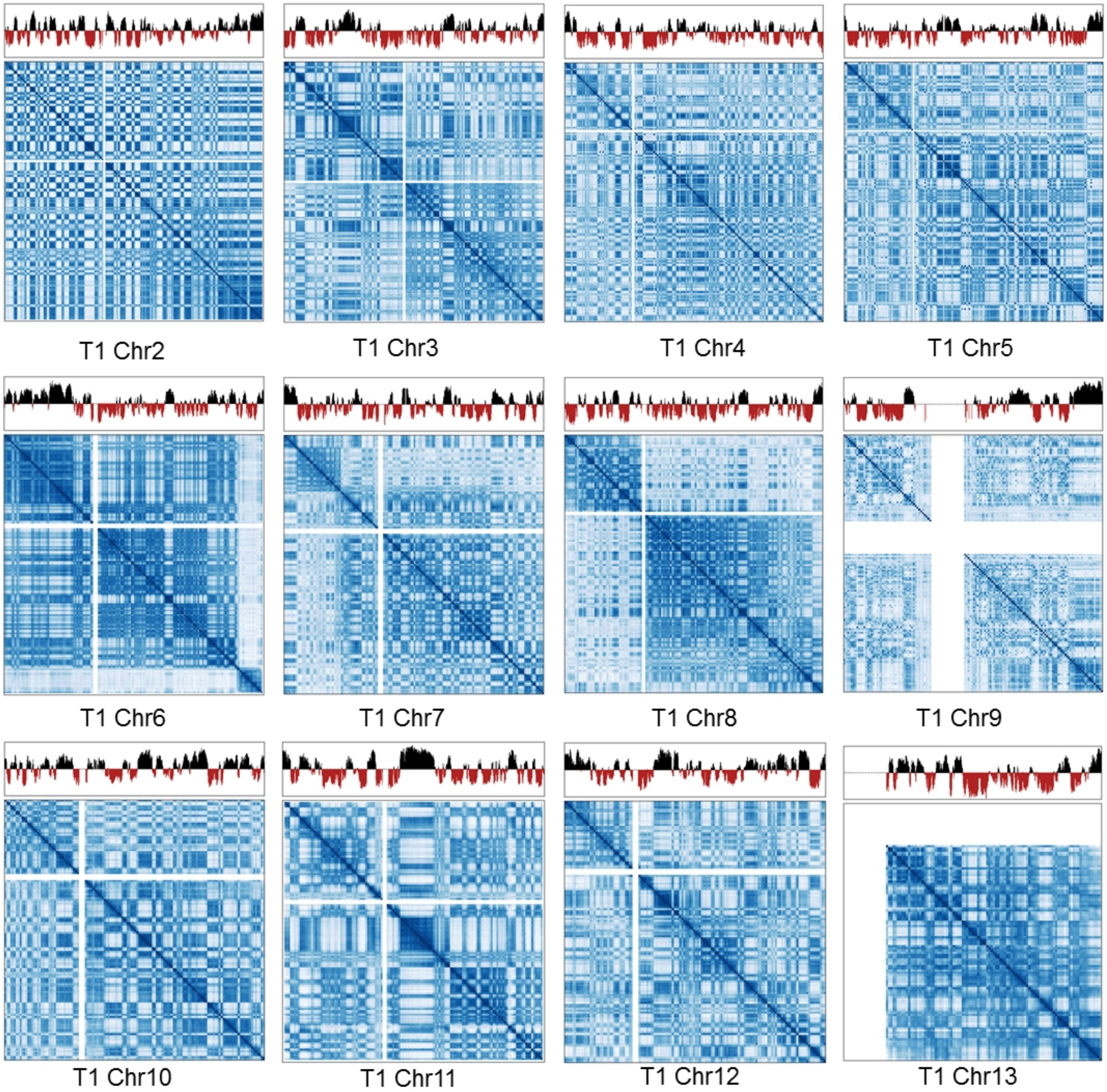

Supplementary Figure 5. Compartment matrix of T1 by chromosome, chr14-X

Compartment matrix and first eigenvectors of T1, chrosome 14 to chrosome X

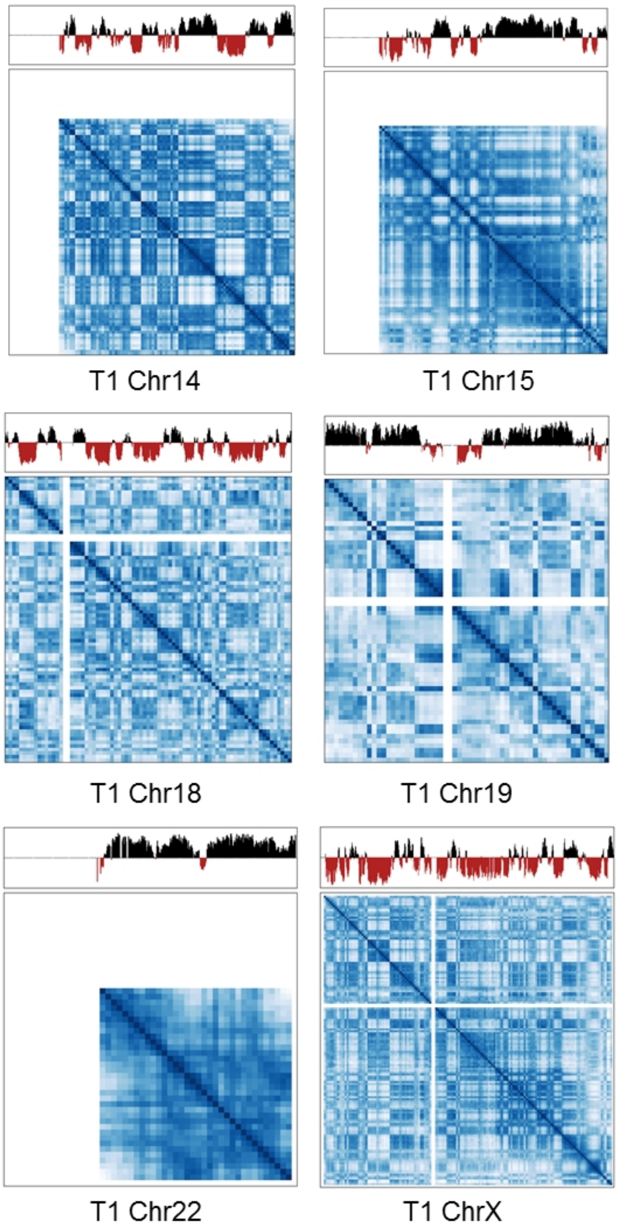

Supplementary Figure 6. Compartment matrix of T4 by chromosome, chr1-12

Compartment matrix and first eigenvectors of T4, chrosome 1 to chrosome 12

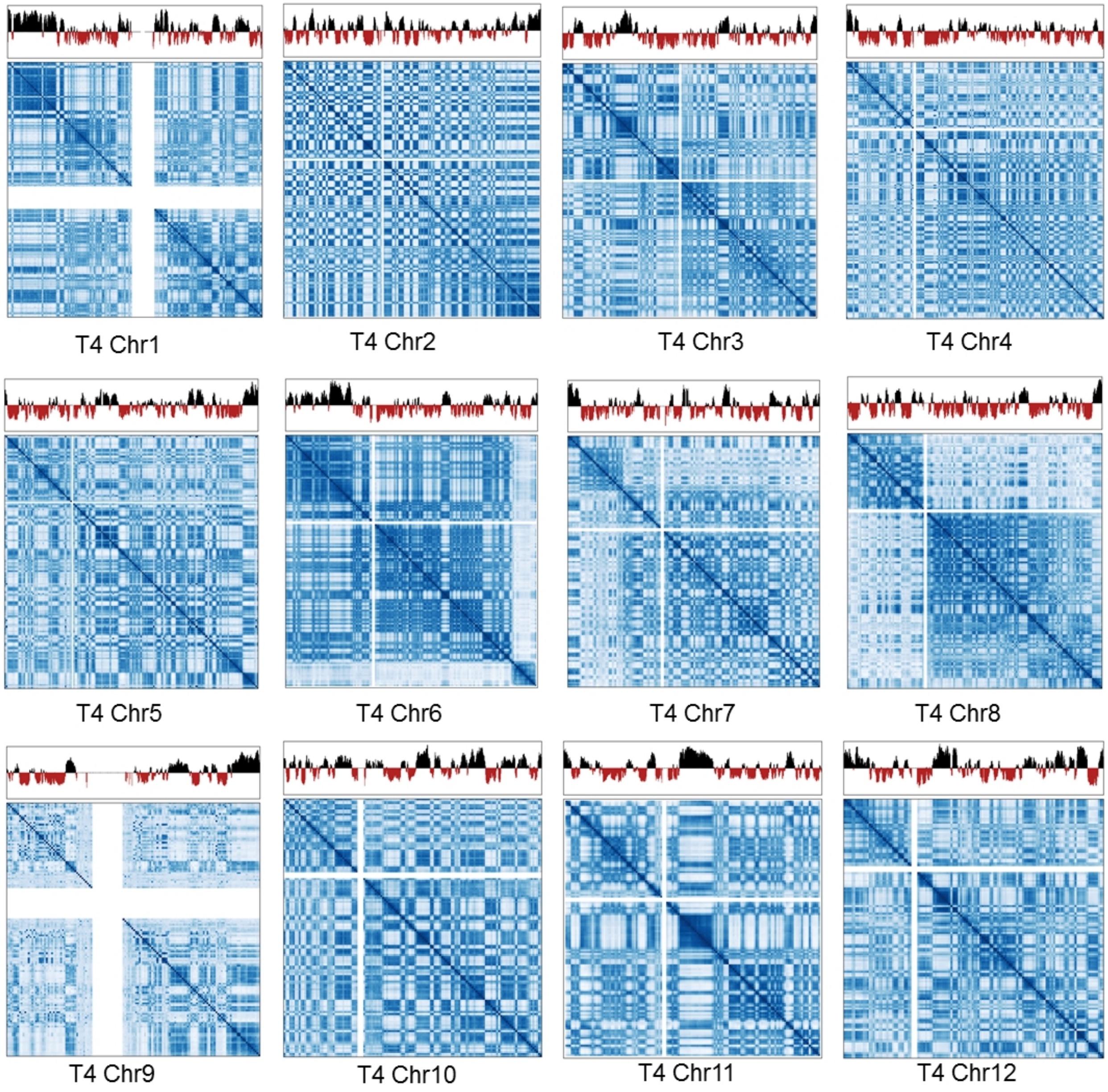

Supplementary Figure 7. Compartment matrix of T4 by chromosome, chr13-X

Compartment matrix and first eigenvectors of T4, chrosome 13 to chrosome X

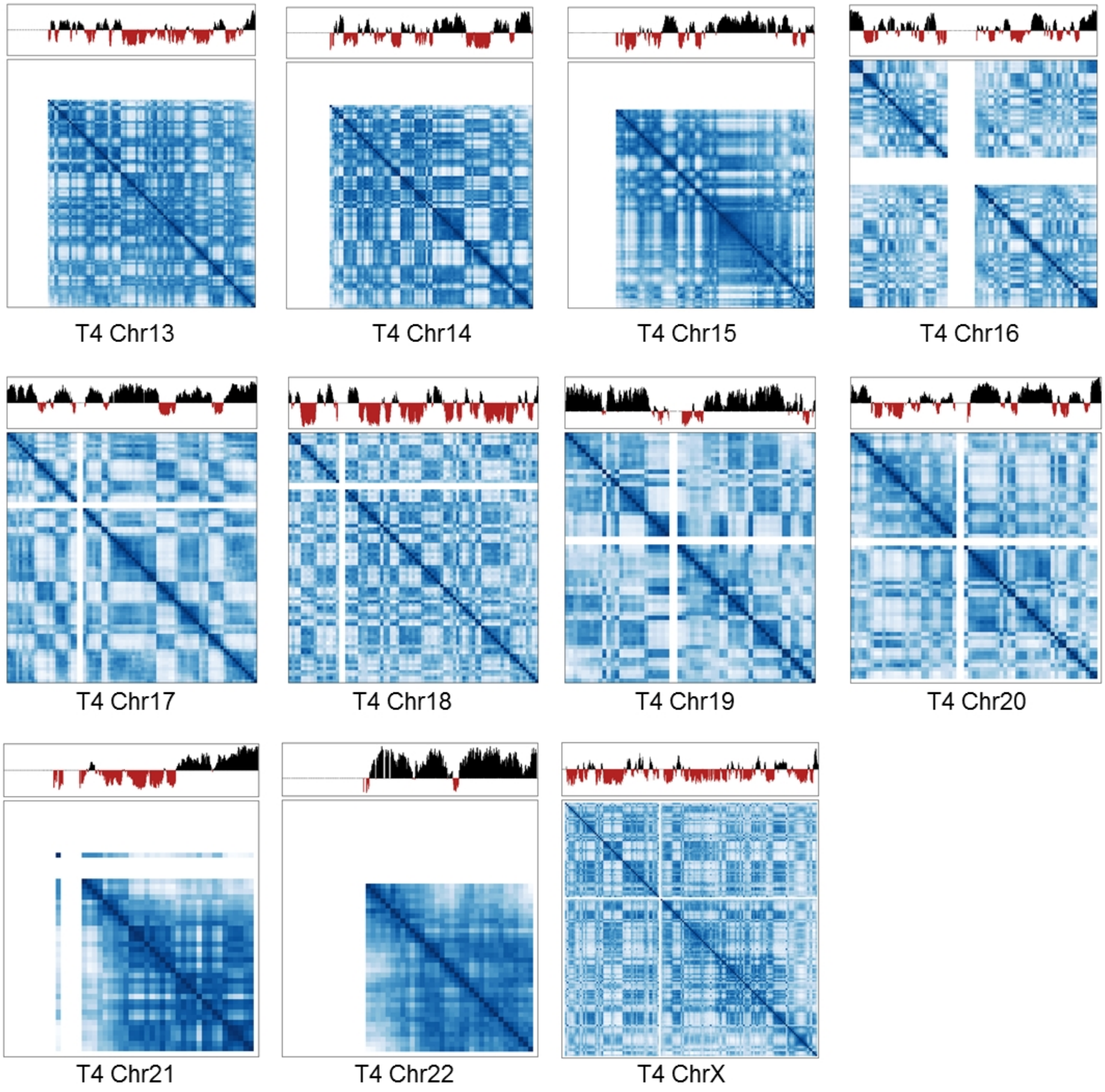

Supplementary Figure 8. Compartment matrix of T16 by chromosome, chr1-12

Compartment matrix and first eigenvectors of T16, chrosome 1 to chrosome 12

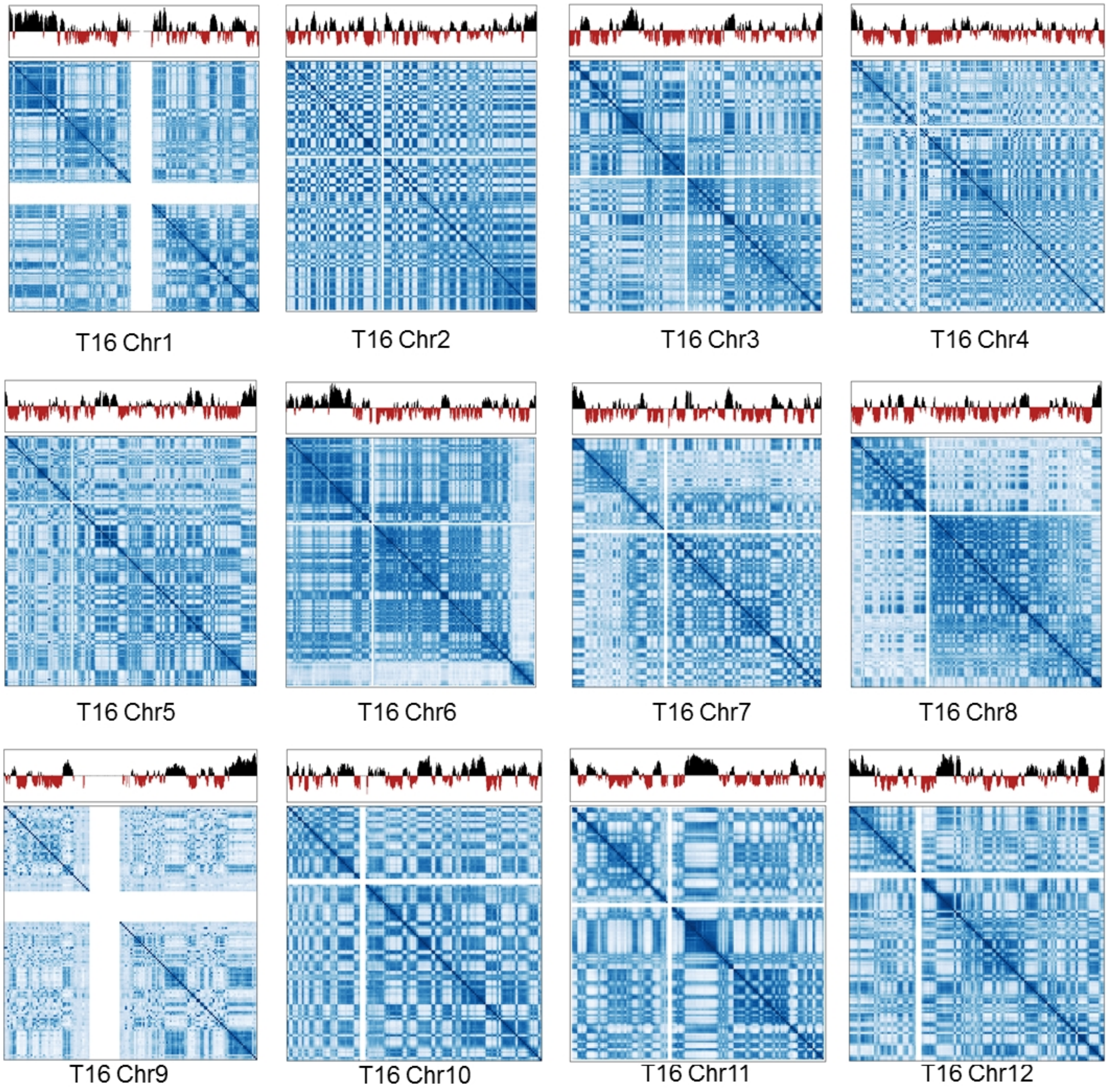

Supplementary Figure 9. Compartment matrix of T16 by chromosome, chr13-X

Compartment matrix and first eigenvectors of T16, chrosome 13 to chrosome X

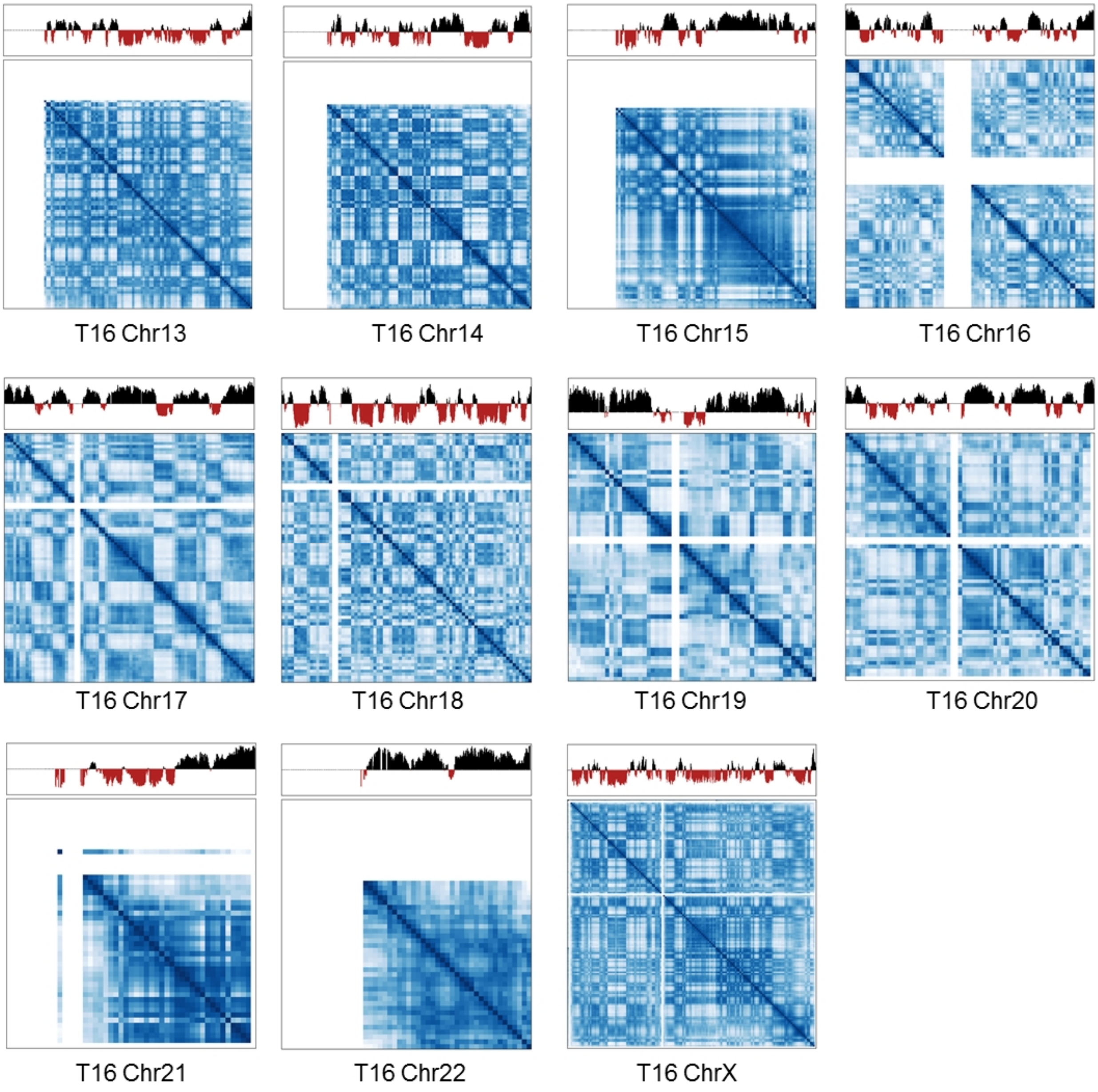

Supplementary Figure 10. Compartment matrix of T24 by chromosome, chr1-12

Compartment matrix and first eigenvectors of T24, chrosome 1 to chrosome 12

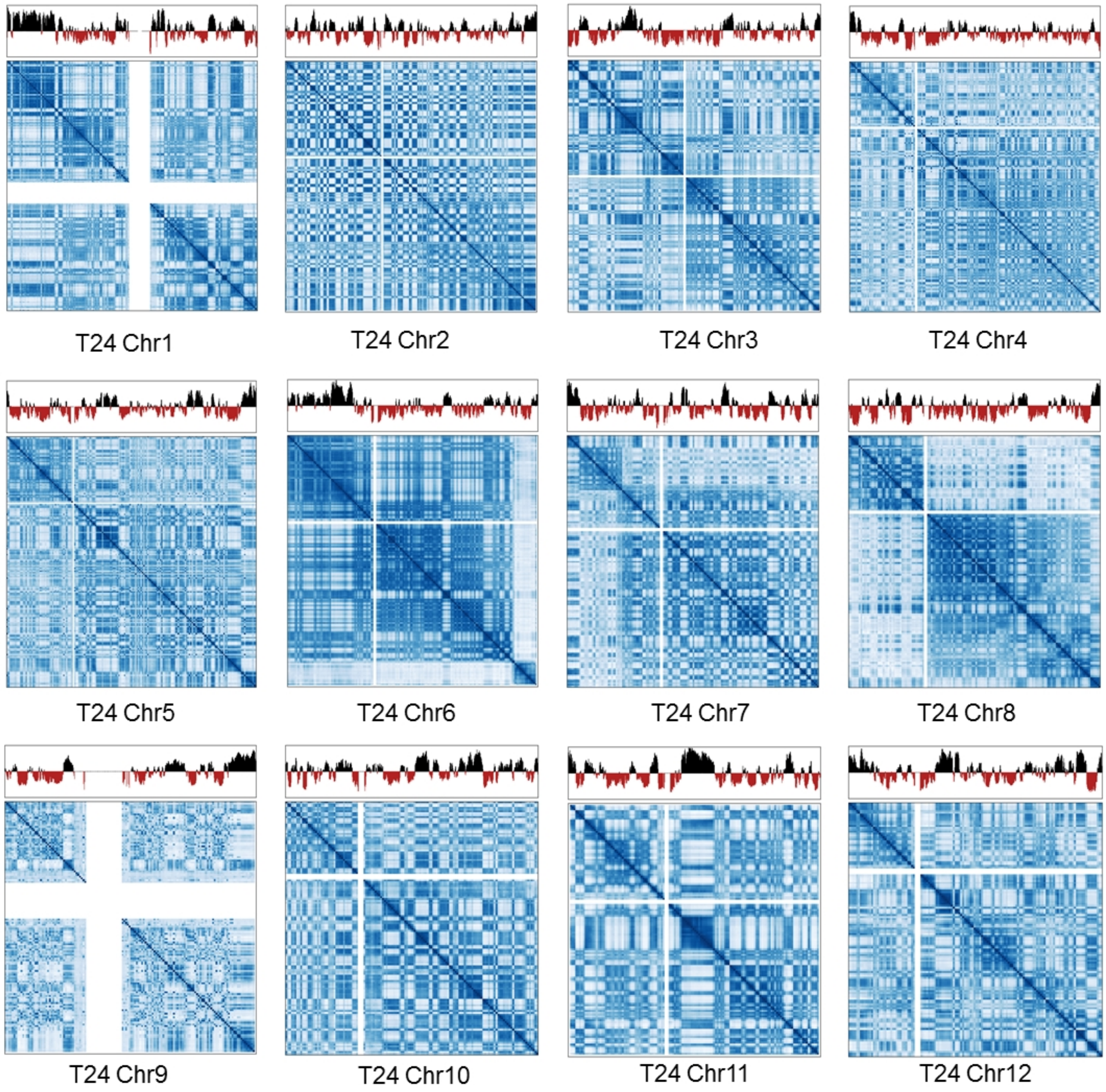

Supplementary Figure 11. Compartment matrix of T24 by chromosome, chr13-X

Compartment matrix and first eigenvectors of T24, chrosome 13 to chrosome X

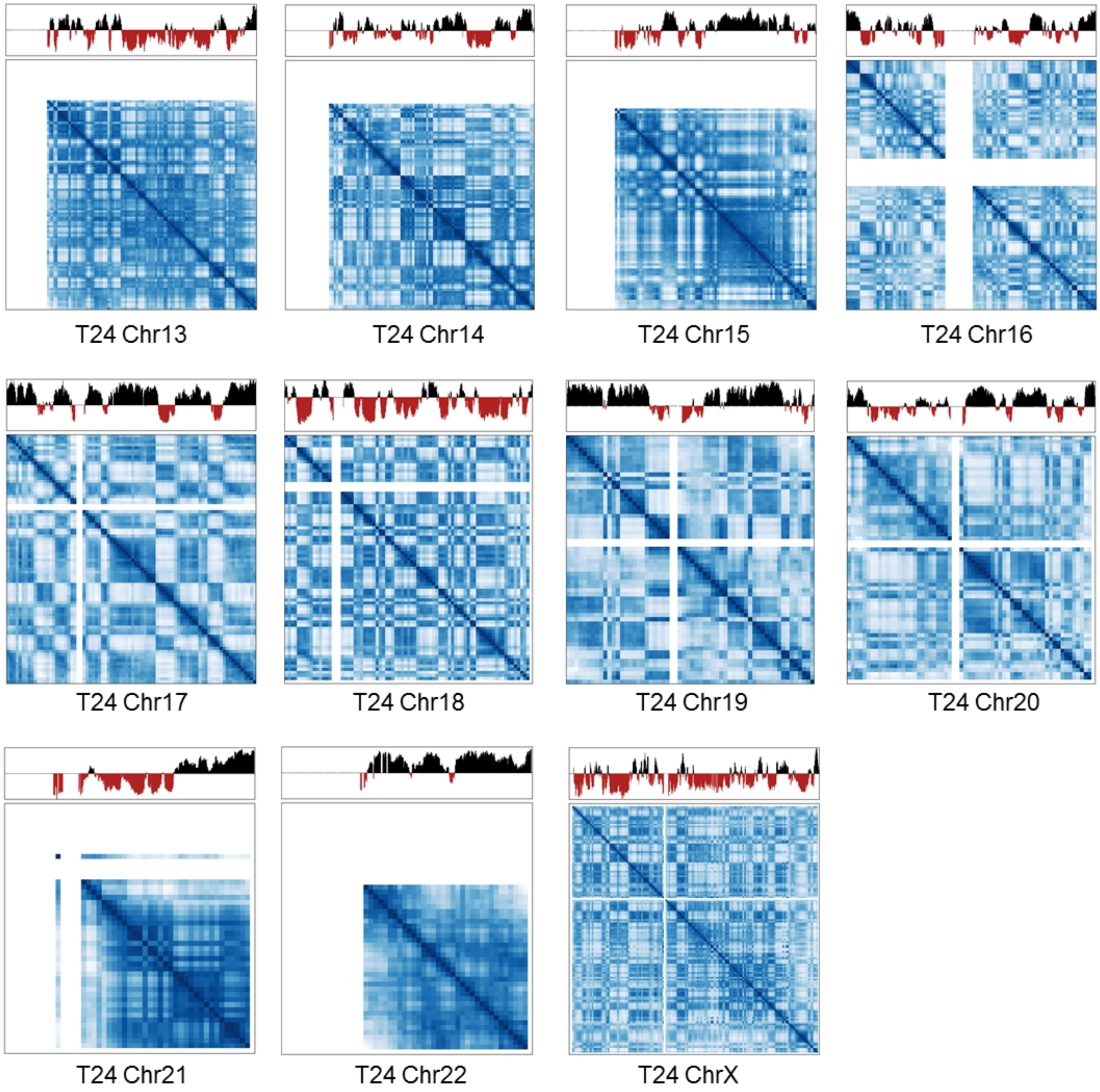

Supplementary Figure 12. Compartment matrix of TamR by chromosome, chr1-12

Compartment matrix and first eigenvectors of TamR, chrosome 1 to chrosome 12

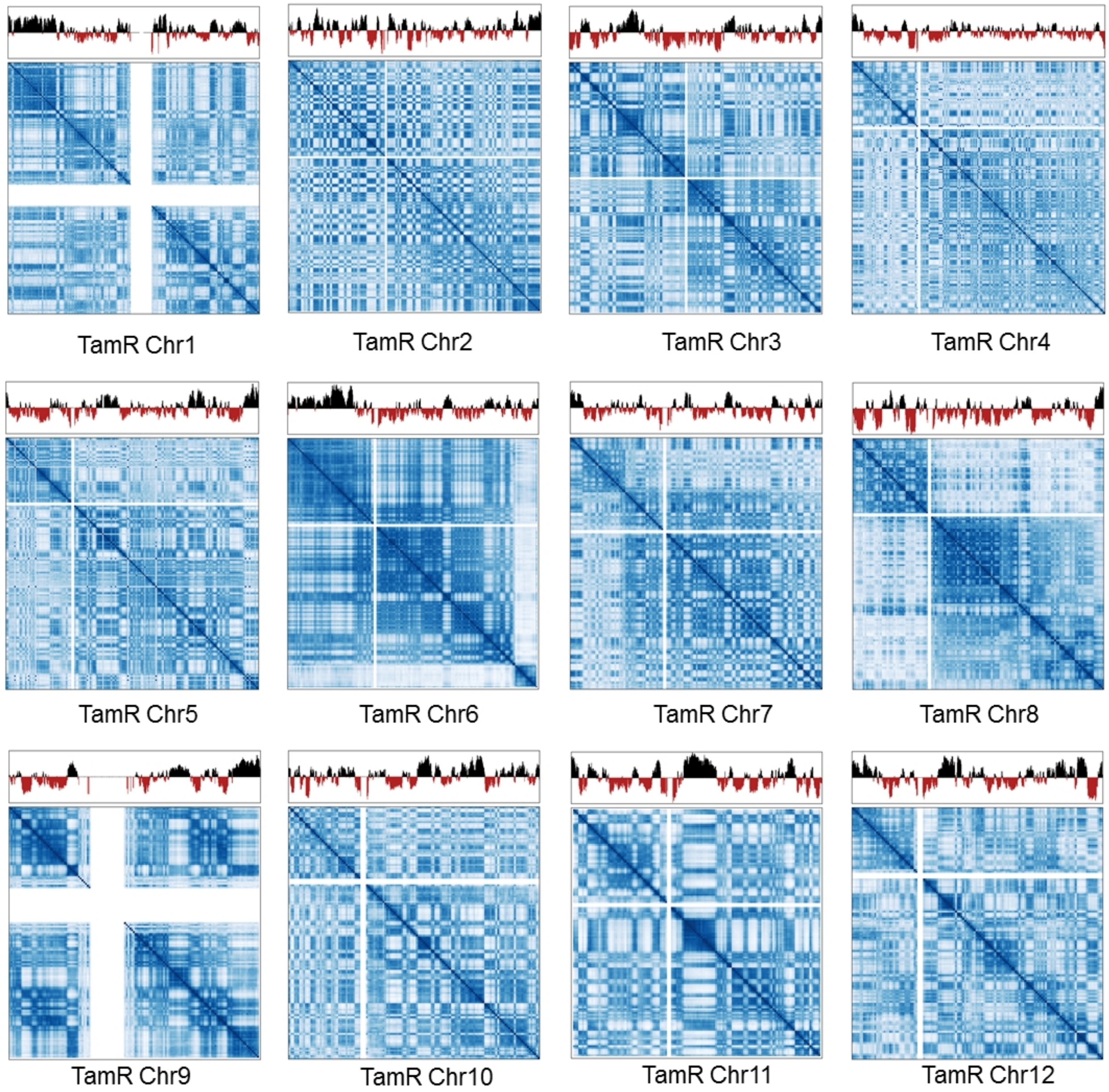

Supplementary Figure 13. Compartment matrix of TamR by chromosome, chr13-X

Compartment matrix and first eigenvectors of TamR, chrosome 13 to chrosome X

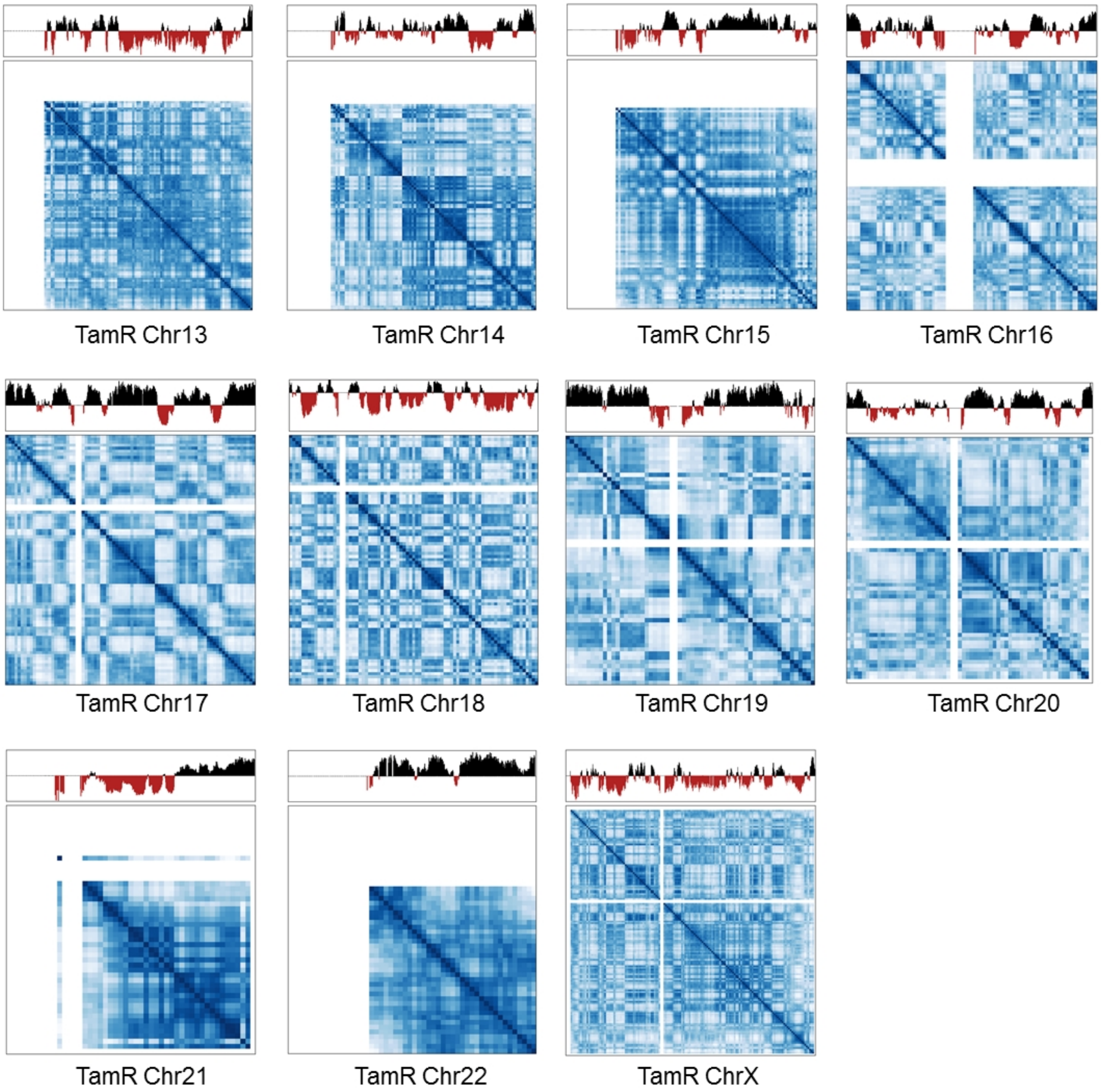

Supplementary Figure 14. Identification of 24 patterns of dynamic compartments

The continuous genomic regions of positive first eigenvector were defined as compartment A (open chromatin) and the coutinuous genomic regions of negative first eigenvector was defined as compartment B (close chromatin). The time serious compartments (A or B individually) were compared as follows:

First, two kinds of compartments: T0 vs T1 Common and T0 vs T1 Transit were identified by comparing of compartments of T0/T1. The Common compartments are the overlapping compartments and the Transit compartments are differential compartments, which will be used in the following steps as well.

Next, T0 vs T1 Common and T0 vs T1 transit were compared with T4, T16, T24 independently to generate the (a)T0 vs T1 Common vs T4/T16/T24 Common, (b)T0 vs T1 Common vs T4/T16/T24 Transit, (c)T0 vs T1 transit vs T4/T16/T24 Common, (d)T0 vs T1 transit vs T4/T16/T24 Transit.

Lastly, the pattern 1-15 were produced by comparing the various time points(T4, T16 and T24) of last step subsets (a, b, c, and d), which are vs T4, vs T16, vs T24 as shown in the Venn diagram. The rest subsets (X shown in Venn diagram) were divided into pattern 16-24 according to the numbers of converted bins (Supplementary Figure 15).

Left column: compartment A, right column: compartment B.

In the Venn diagram, numbers without parenthesis are the numbers of compartments, numbers with parenthesis are the patterns.

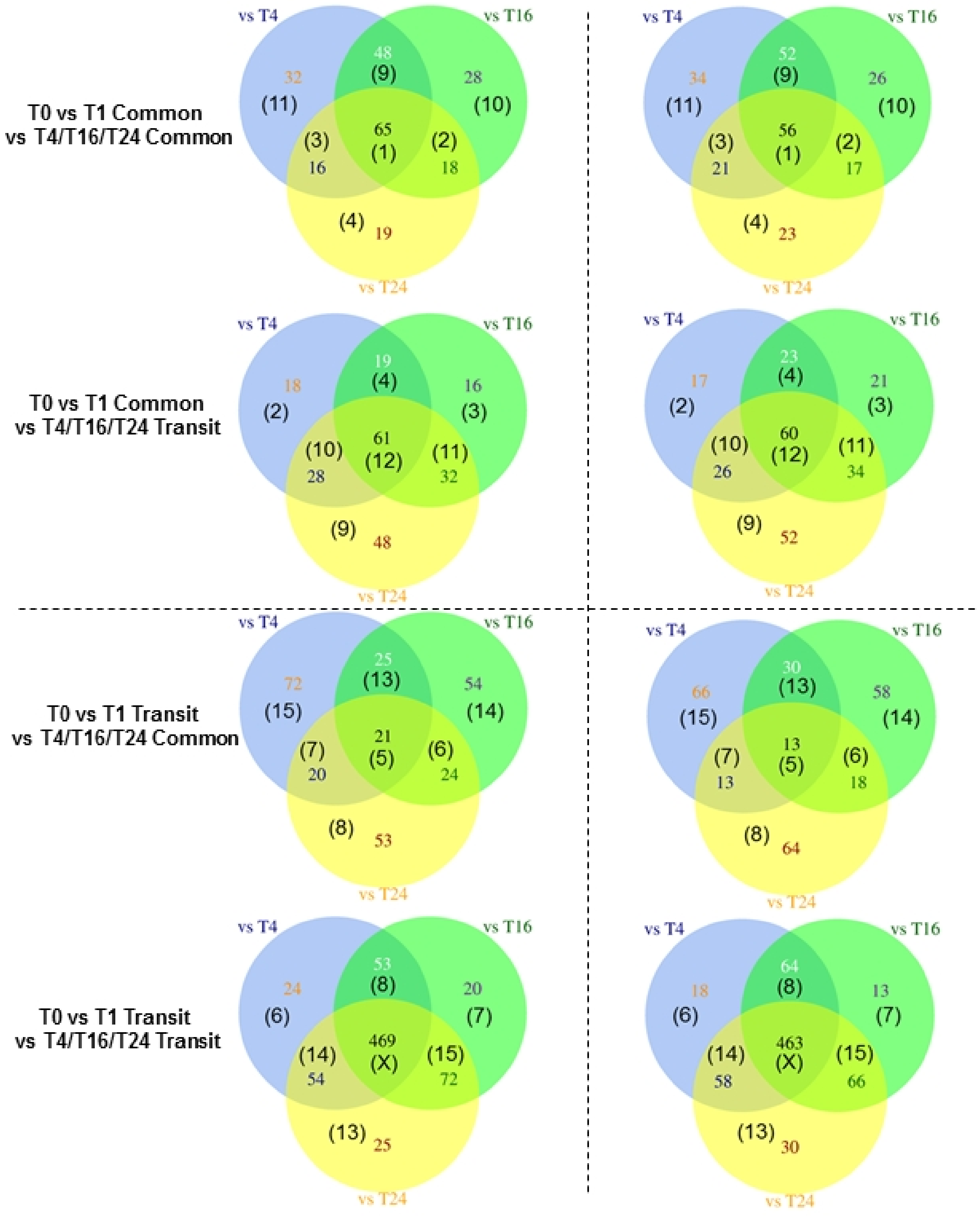
T0 vs T1 Common
vs T4/T16/T24 Common
vs T4
vs T16
vs T24
32
(11)
48
(9)
28
(10)
(3)
16
65
(1)
(2)
18
(4)
19
34
(11)
52
(9)
26
(10)
(3)
21
56
(1)
(2)
17
(4)
23
T0 vs T1 Common
vs T4/T16/T24 Transit
18
(2)
19
(4)
16
(3)
(10)
28
61
(12)
(11)
32
(9)
48
17
(2)
23
(4)
21
(3)
(10)
26
60
(12)
(11)
34
(9)
52
T0 vs T1 Transit
vs T4/T16/T24 Common
72
(15)
25
(13)
54
(14)
(7)
20
21
(5)
(6)
24
(8)
53
66
(15)
30
(13)
58
(14)
(7)
13
13
(5)
(6)
18
(8)
64
T0 vs T1 Transit
vs T4/T16/T24 Transit
24
(6)
53
(8)
20
(7)
(14)
54
469
(X)
(15)
72
(13)
25
18
(6)
64
(8)
13
(7)
(14)
58
463
(X)
(15)
66
(13)
30

Supplementary Figure 15. Definition of Converted bins

The converted bins is defined as follows:

The bin size of compartment is 100K. The conversion of positive first eigenvector of compartment to negative first eigenvector of compartment or the negative first eigenvector of compartment to the positive first eigenvector of compartment at the same genome loci is defined as converted bin.

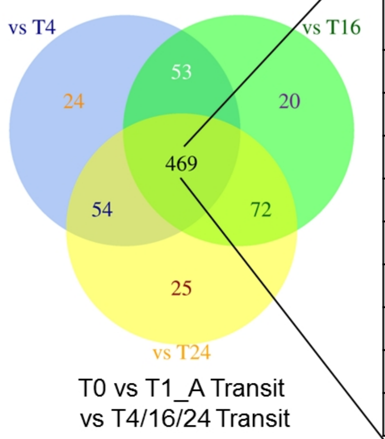

Converted bins			Com Num	Pattern
vs T4	vs T16	vs T24		
0	0	0	26	(16)
0	0	≥1	22	(17)
0	≥1	0	9	(18)
0	≥1	≥1	30	(19)
≥1	0	0	10	(20)
≥1	0	≥1	27	(21)
≥1	≥1	0	41	(22)
Any ≥1 and <3			189	(23)
Any ≥ 3			115	(24)

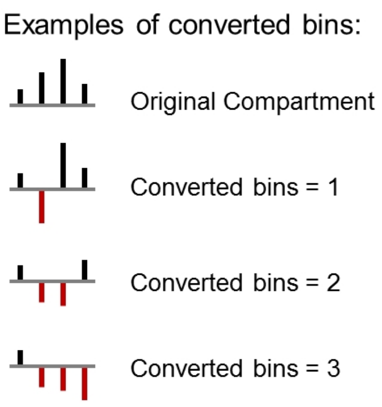

Com Num: Compartment number.

Supplementary Figure 16. The variance of first eigenvectors of compartment types in T0, T1, T4, T16, T24 and TamR

For each 100K bin, the variance of first eigenvectors of compartment in T0, T1, T4, T16, T24 and TamR was calculated. Then the variance (σ^2) of each bin of the compartment in various compartment types was averaged. The p value was determined by two-sided Wilcoxon rank-sum test.

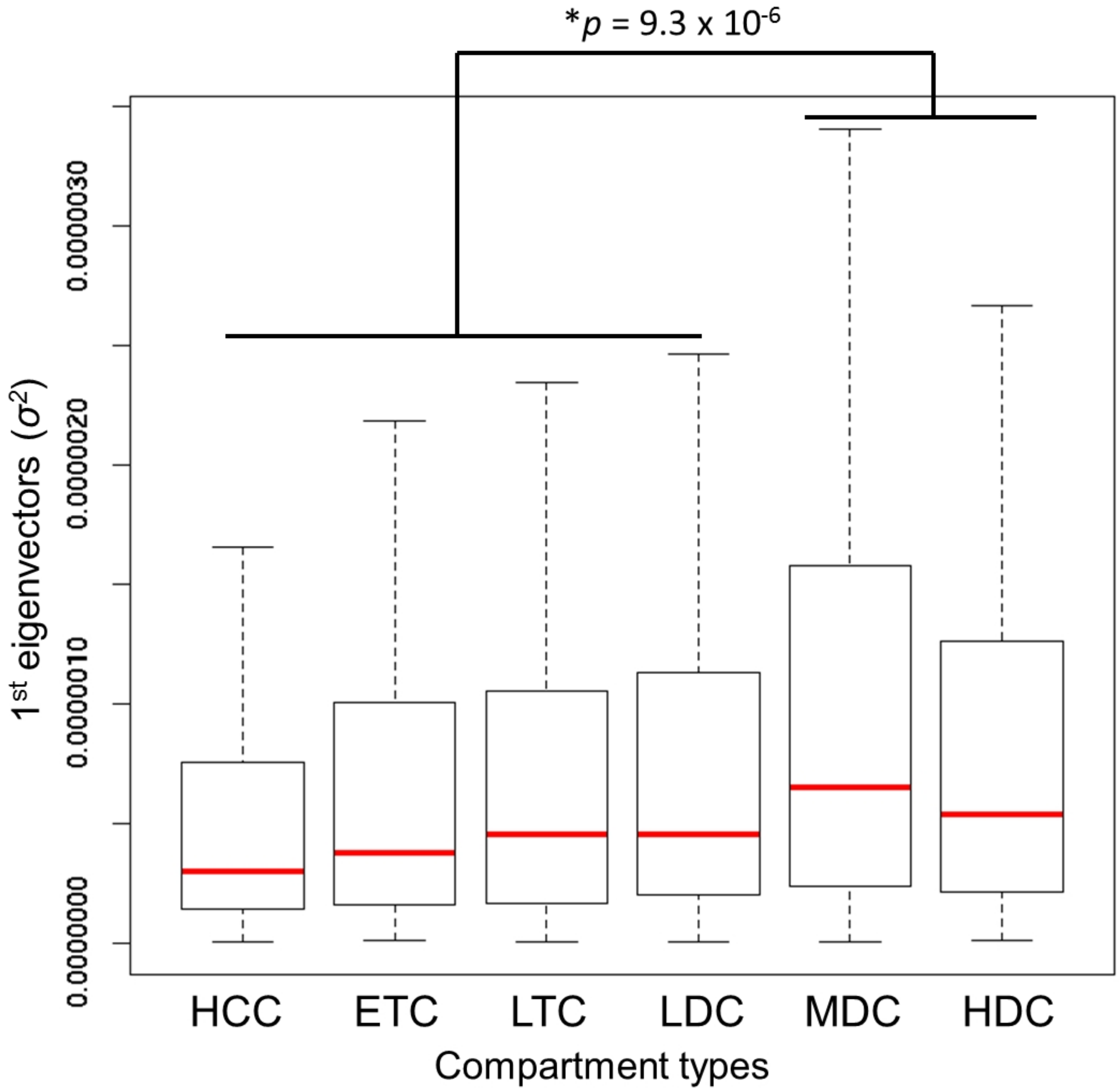

Supplementary Figure 17. Differential binding analysis of ERα in tamoxifen resistant altered compartments

ERα differential peaks (DPs) were identified with DiffBind[2,3] using TamR vs. T0/T1/T4/T16/T24 as the contrast. The numbers of DPs in each temporal dynamic re-compartmentalization (TDRC) was calculated, among them the percentage of DPs within tamoxifen resistant altered compartment (TRAC) was demonstrated as figure.

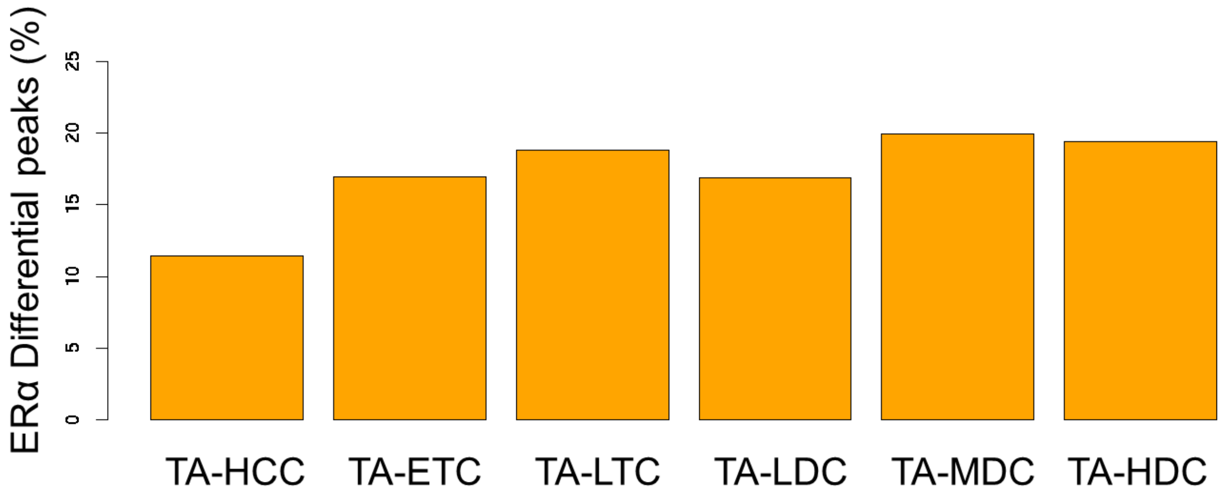

Supplementary Figure 18. The percentage heatmap of E2-induced MCF7 compartment A with ERα peaks

Number 1-24 is the pattern name.

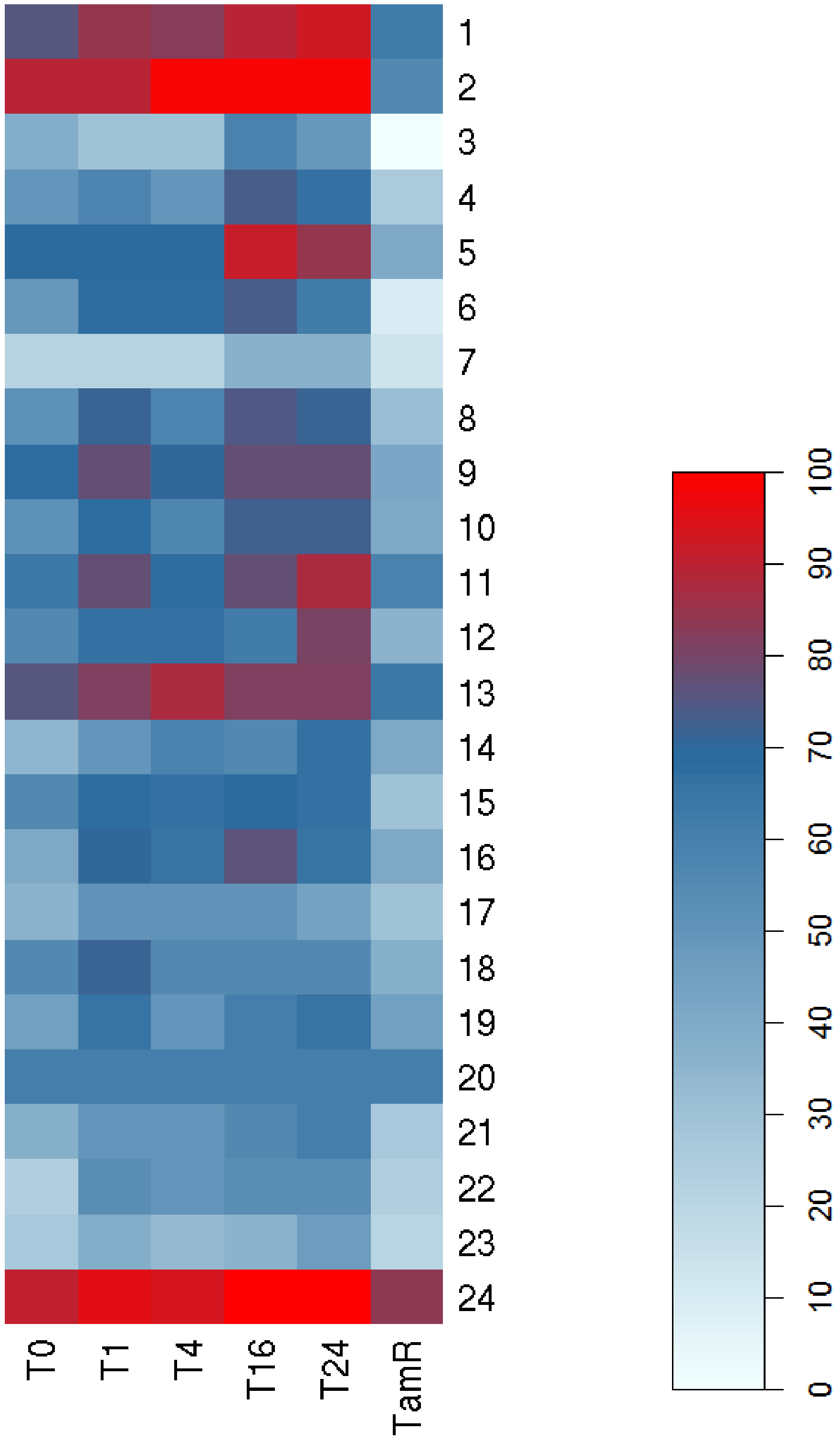

Supplementary Figure 19. The percentage heatmap of E2-induced MCF7 compartment B with ERα peaks

Number 1-24 is the pattern name.

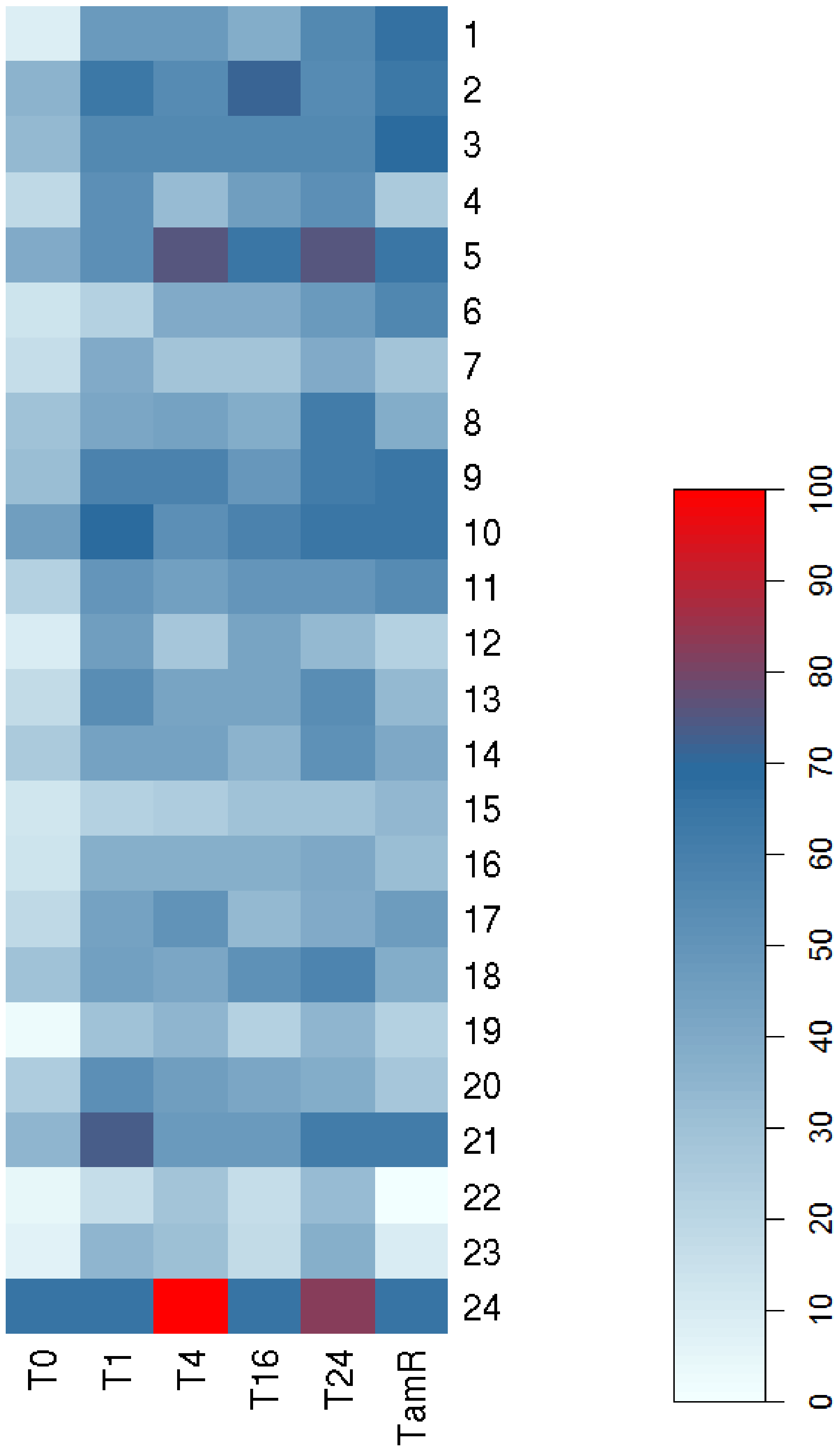

Supplementary Figure 20. Validation of 3C-qPCR for HOMER loops

HOMER interaction loops are named as genes located at the interaction loops. Three 3C-qPCRs were performed for each loop. The MCF7L TamR group was compared with MCF7 or MCF7Lp by ANOVA analysis. Error bars represent standard error of the mean (S.E.M.) with three experiments.

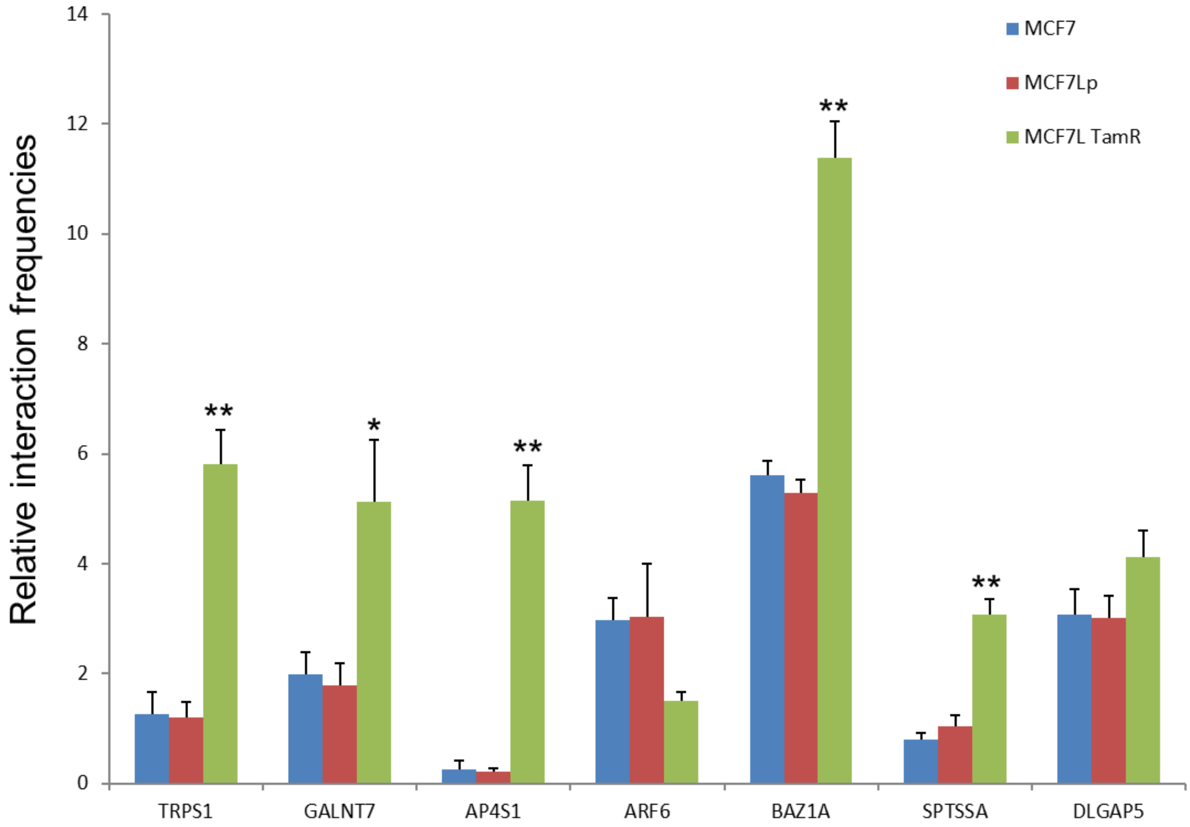

MCF7: MCF7 cell lines

MCF7Lp: MCF7L parental cells

MCF7L TamR: MCF7L Tamoxifen resistant cells

*: $p < 0.05$; **: $p < 0.01$, two-sided t-test

Supplementary Figure 21. The enrichment of KEGG pathways for 1747 genes in T1/T0 differential compartments

The differential compartments were defined as at least one converted bin (refer to Supplementary Figure 15) between two compared compartments. The differentially expressed 1,747 genes of TamR vs T0 in the differential compartments of T1 vs T0 were identified. These genes were then enriched with KEGG pathways using GSEA[4].

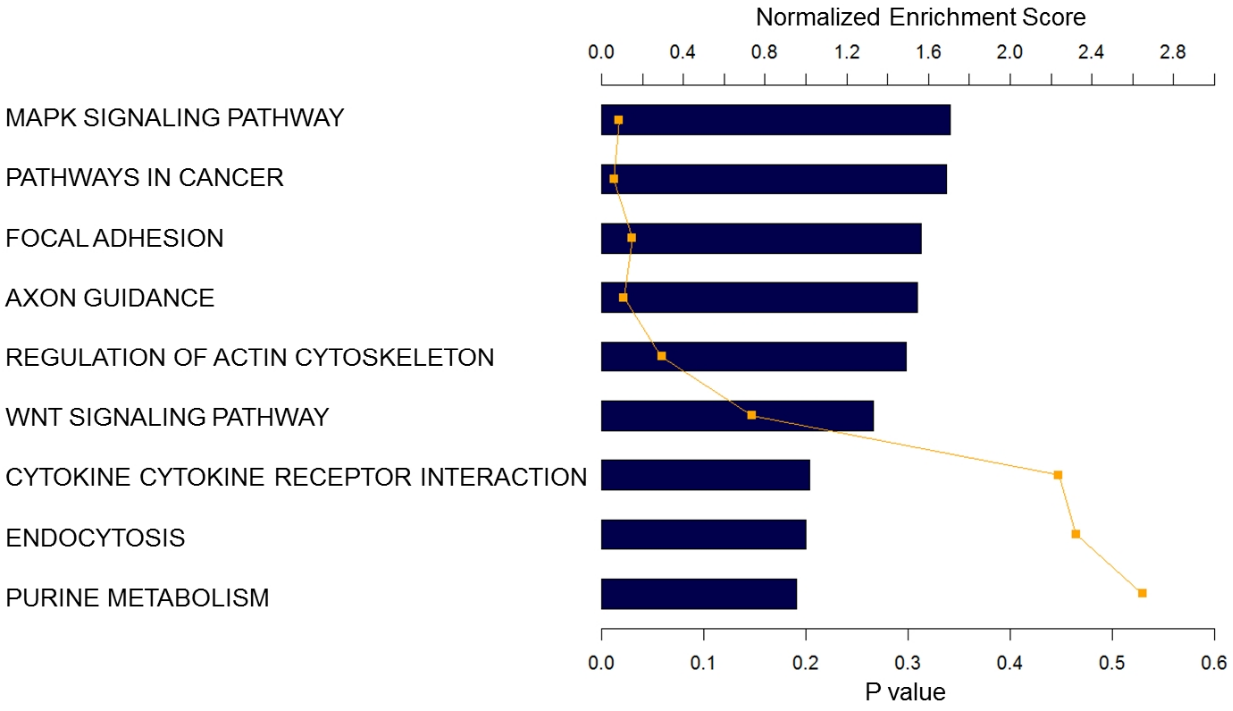

Supplementary Figure 22. The enrichment of KEGG pathways for 1397 genes in T4/T0 differential compartments

The differential compartments were defined as at least one converted bin (refer to Supplementary Figure 15) between two compared compartments. The differentially expressed 1,397 genes of TamR vs T0 in the differential compartments of T4 vs T0 were identified. These genes were then enriched with KEGG pathways using GSEA[4].

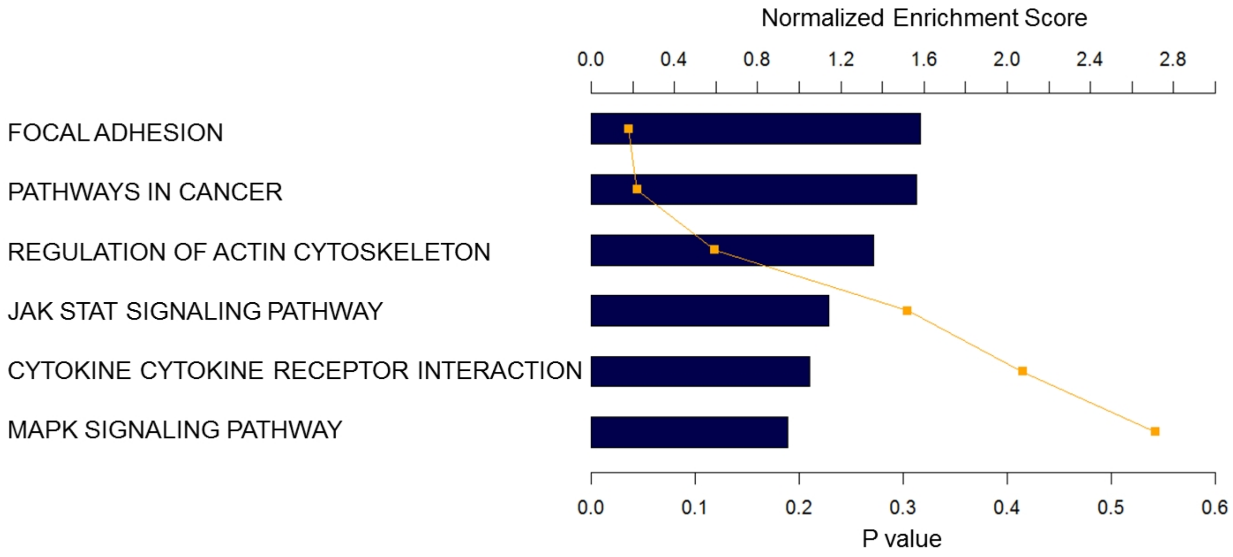

Supplementary Figure 23. The enrichment of KEGG pathways for 1,514 genes in T16/T0 differential compartments

The differential compartments were defined as at least one converted bin (refer to Supplementary Figure 15) between two compared compartments. The differentially expressed 1,514 genes of TamR vs T0 in the differential compartments of T16 vs T0 were identified. These genes were then enriched with KEGG pathways using GSEA[4]. 2005).

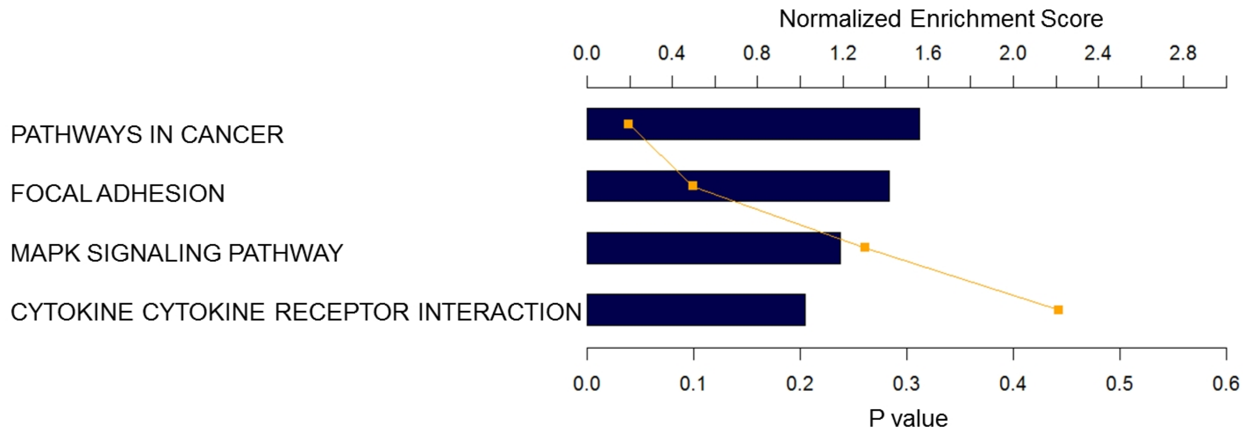

Supplementary Figure 24. The enrichment of KEGG pathways for 2,023 genes in T24/T0 differential compartments

The differential compartments were defined as at least one converted bin (refer to Supplementary Figure 15) between two compared compartments. The differentially expressed 2,023 genes of TamR vs T0 in the differential compartments of T24 vs T0 were identified. These genes were then enriched with KEGG pathways using GSEA[4].

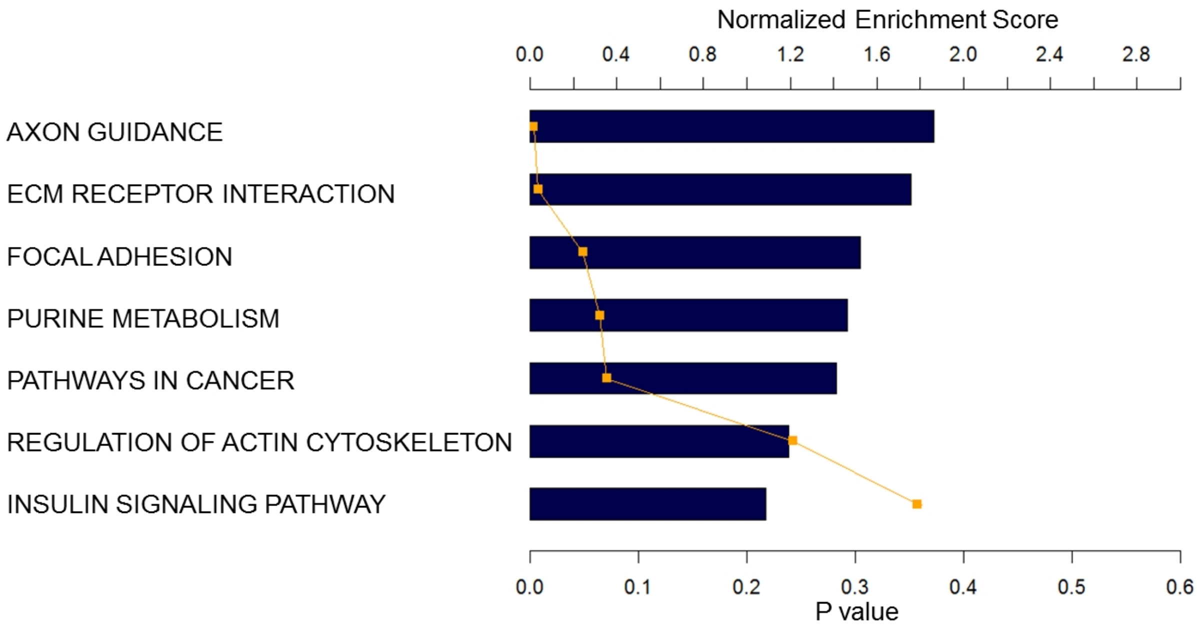

Supplementary Figure 25. The enrichment of KEGG pathways for 2,185 genes in TamR/T0 differential compartments

The differential compartments were defined as at least one converted bin (refer to Supplementary Figure 15) between two compared compartments. The differentially expressed 2,185 genes of TamR vs T0 in the differential compartments of TamR vs T0 were identified. These genes were then enriched with KEGG pathways using GSEA[4].

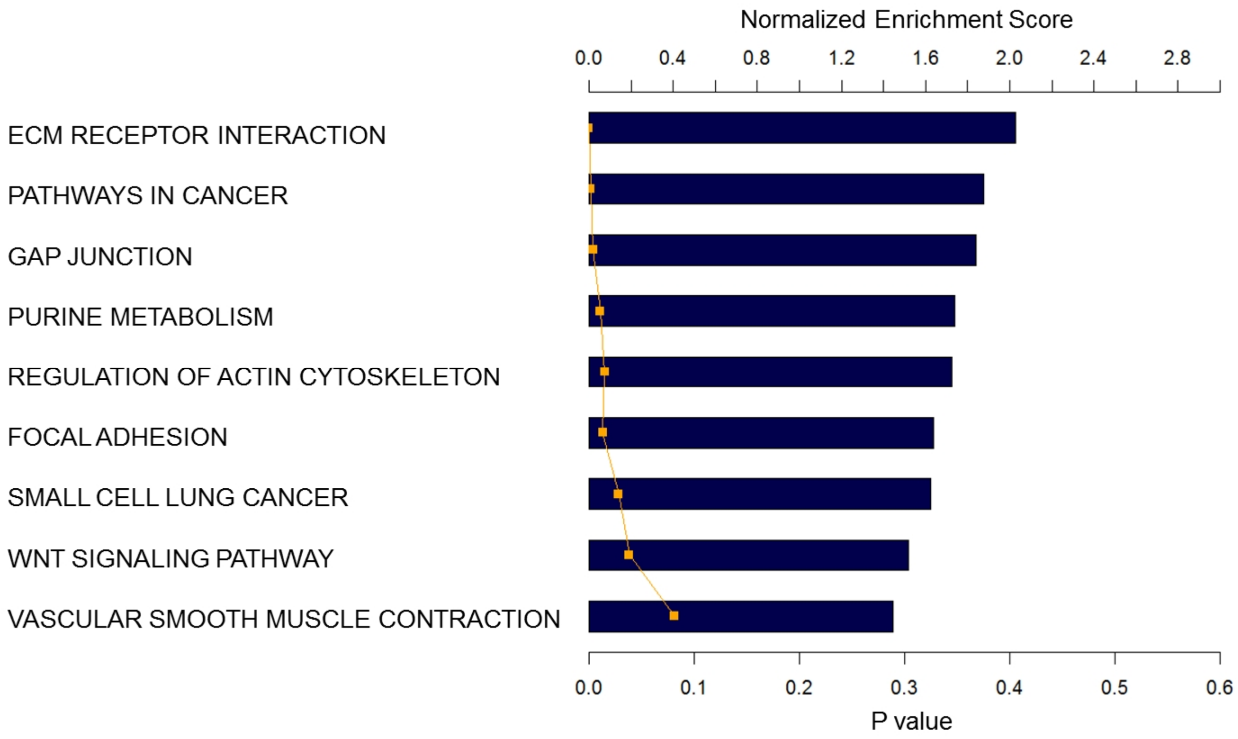

Supplementary Figure 26. Relapse-free survival analysis of SOX4

Kaplan-Meier plot of SOX4 was generated with the probability of relapse-free survival in ER+ patients receiving only Tamoxifen but without chemotherapy (n = 670), who were stratified by SOX4 mRNA levels at the top quartile (25%) vs. the rest (75%). *p* value was computed with the log-rank test. Analysis was referred to the published paper[5].

HR: Hazard Ratio

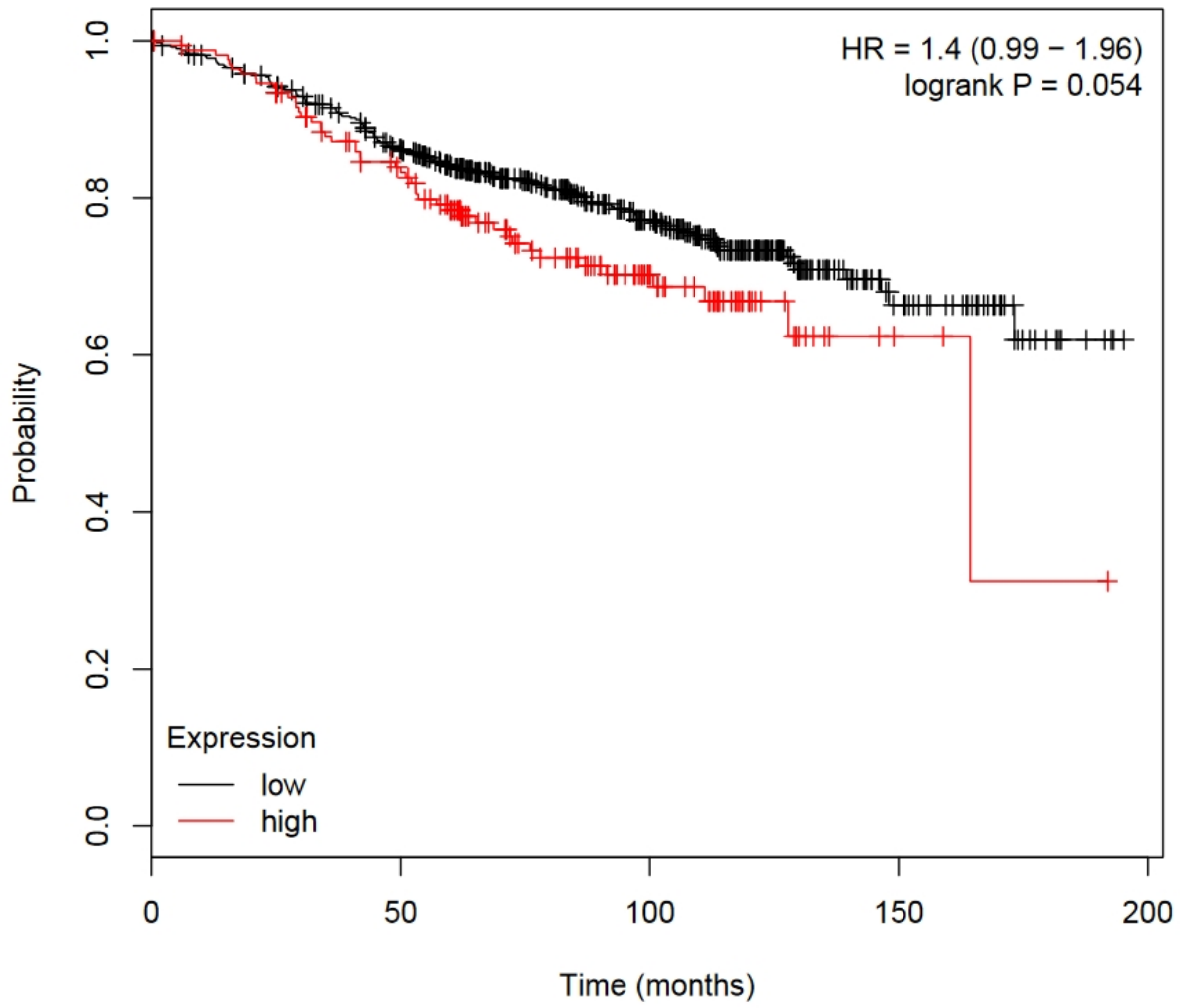

Supplementary Figure 27. Relapse-free survival analysis of GPRC5C

Kaplan-Meier plot of GPRC5C was generated with the probability of relapse-free survival in ER+ patients receiving only Tamoxifen but without chemotherapy (n = 670), who were stratified by GPRC5C mRNA levels at the top quartile (25%) vs. the rest (75%). *p* value was computed with the log-rank test. Analysis was referred to the published paper[5].

HR: Hazard Ratio

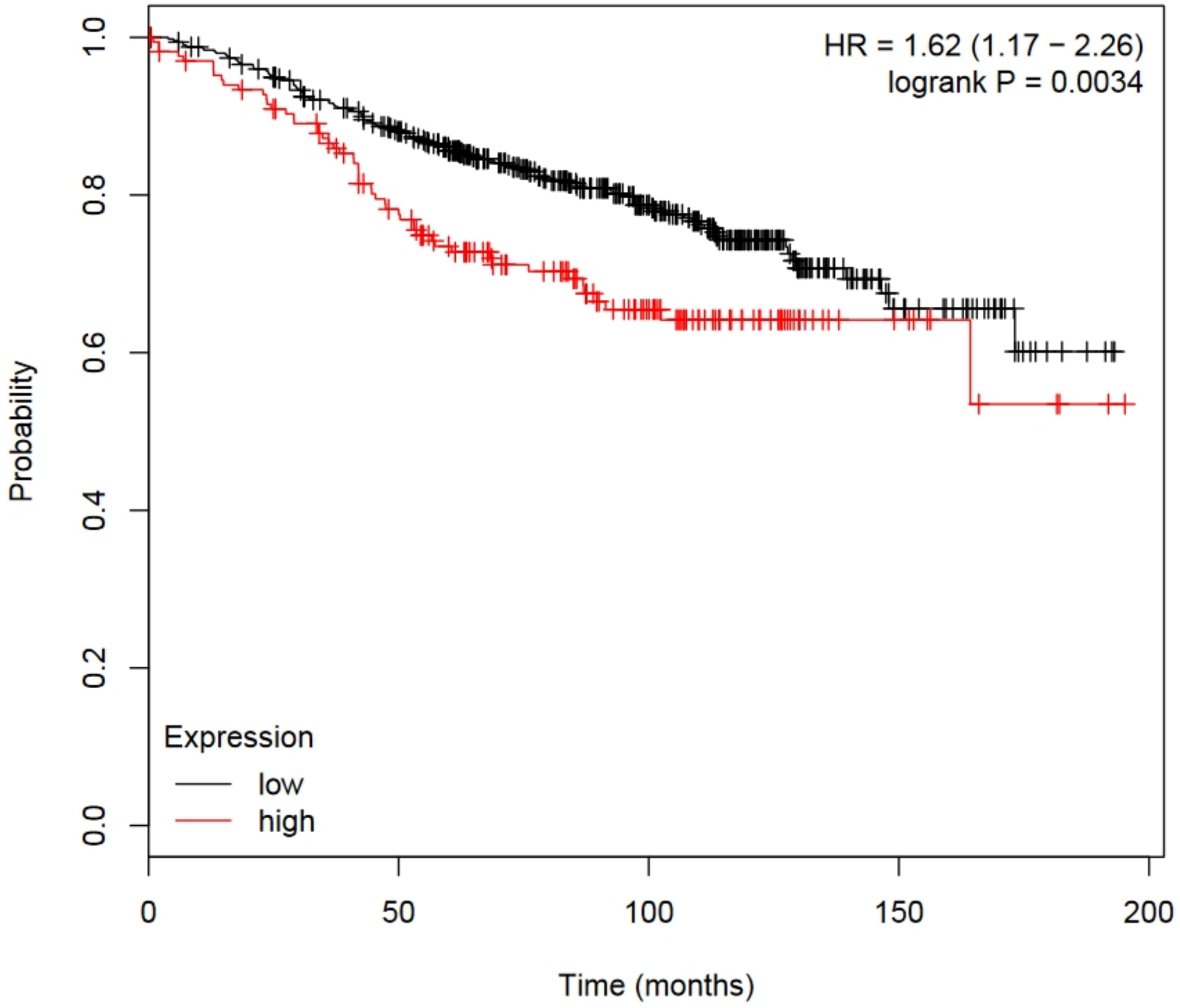

Supplementary Figure 28. Relapse-free survival analysis of PRC1

Kaplan-Meier plot of PRC1 was generated with the probability of relapse-free survival in ER+ patients receiving only Tamoxifen but without chemotherapy (n = 670), who were stratified by PRC1 mRNA levels at the top quartile (25%) vs. the rest (75%). *p* value was computed with the log-rank test. Analysis was referred to the published paper[5].

HR: Hazard Ratio

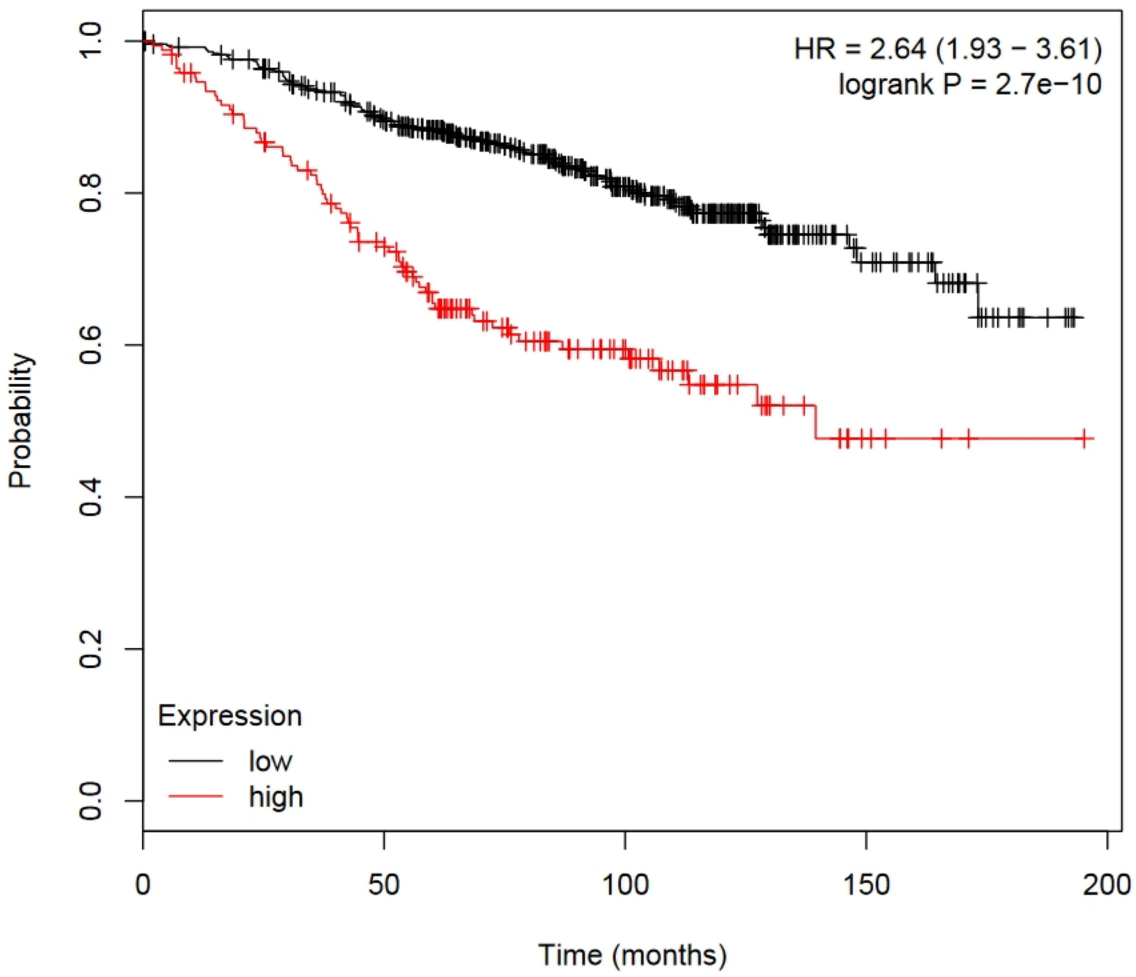

Supplementary Figure 29. 256 combinations of E2-induced time-dependent compartments

Using T0 as a contrast, there are four combinations of compartment changes from T0 to T1: A→A, B→B, A→B, and B→A, same can be obtained for T4, T16 and T24 respectively. Thus, the total combinations of possible E2-induced time-dependent compartments are 4 X 4 X 4 X 4 = 256.

	1	2	3	4
T0 → T1	A → A	B → B	A → B	B → A
T0 → T4	A → A	B → B	A → B	B → A
T0 → T16	A → A	B → B	A → B	B → A
T0 → T24	A → A	B → B	A → B	B → A

Supplementary Figure 30. The same trends of E2-induced time-dependent compartments in MCF7 and T47D cell lines

Despite that there are 256 combinations of E2-induced time-dependent compartments shown in Supplementary Figure 30, for the simplicity, we just consider five trends, including two unchanged compartments (A→A, B→B in all time points), two flipped compartments (A→B, and B→A in all time points), and miscellaneous (dynamic changed) compartments which combines all other dynamic changes. As shown in the figure below, the major trend is miscellaneous compartments in both MCF7 and T47D cell lines.

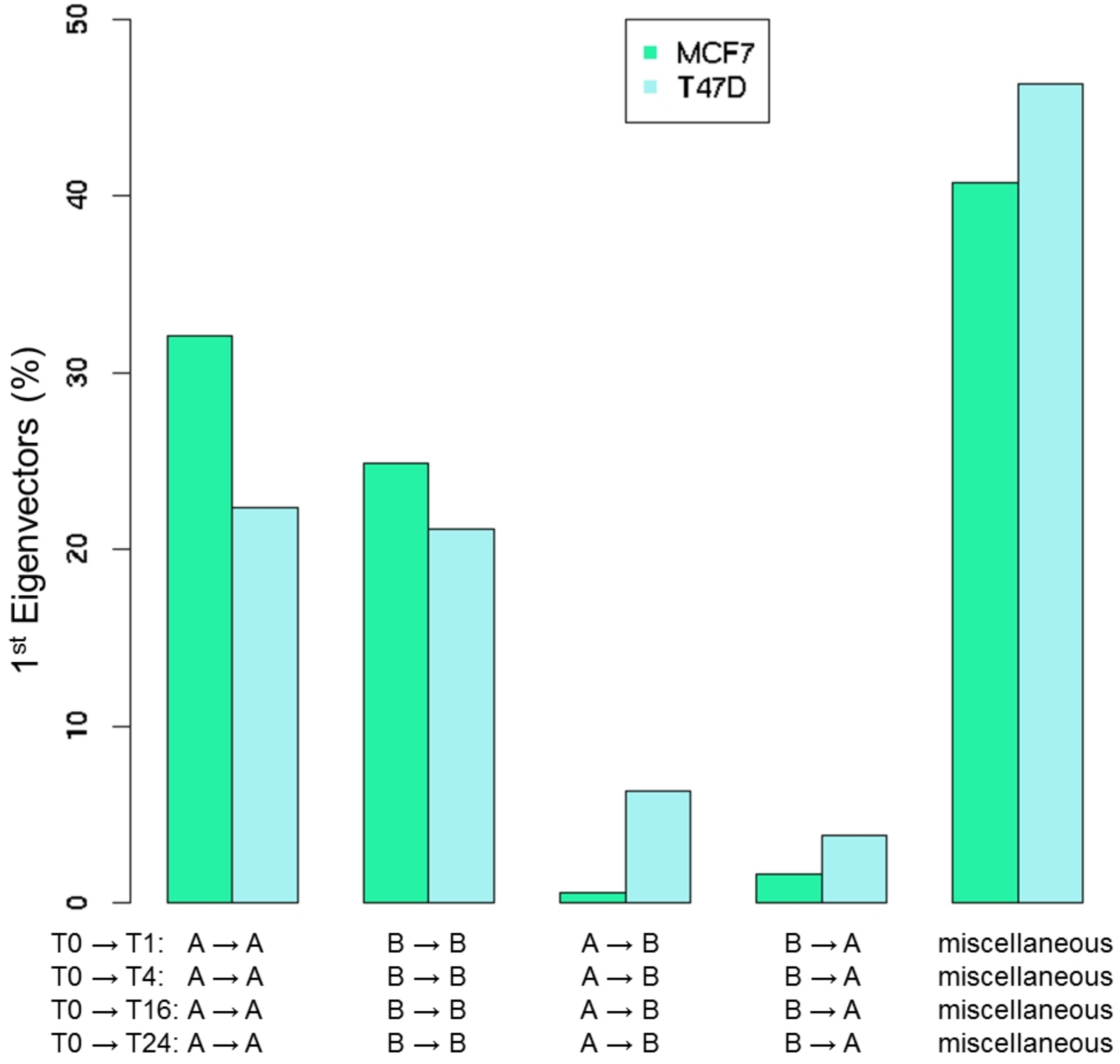

Supplementary Figure 31. The heatmap showing MCF7-TamR altered compartments in comparison to E2-induced time-dependent compartments

There are four possibilities of compartment changes from MCF7 T0 to MCF7-TamR cells: A→A, B→B, A→B, and B→A. The following heatmap showed the percentage of all possible altered compartments in MCF7-TamR and their overlaps with E2-induced time-dependent compartments.

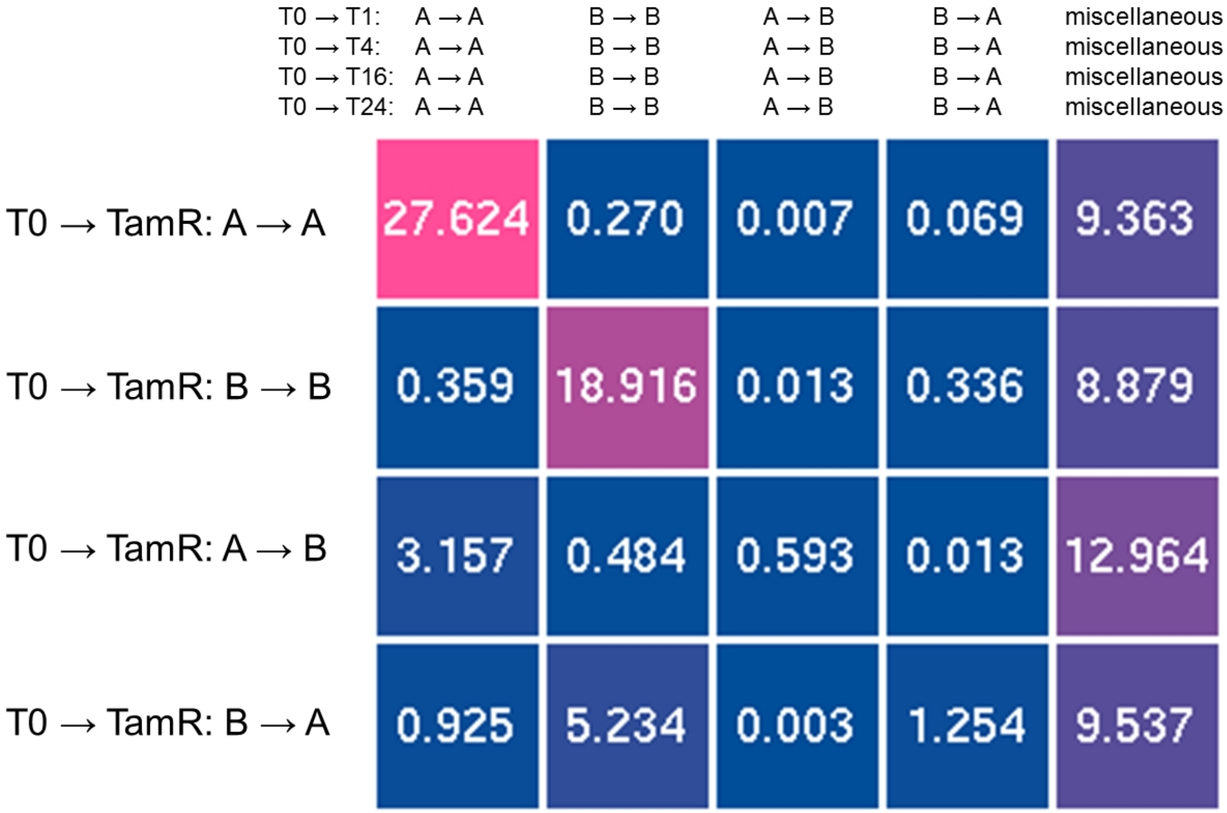

Supplementary Figure 32. The heatmap showing T47D-TamR altered compartments in comparison to E2-induced time-dependent compartments

There are four possibilities of compartment changes from T47D T0 to T47D-TamR cells: A to A, B to B, A to B and B to A. A→A, B→B, A→B, and B→A. The following heatmap showed the percentage of all possible altered compartments in T47D-TamR and their overlaps with E2-induced time-dependent compartments.

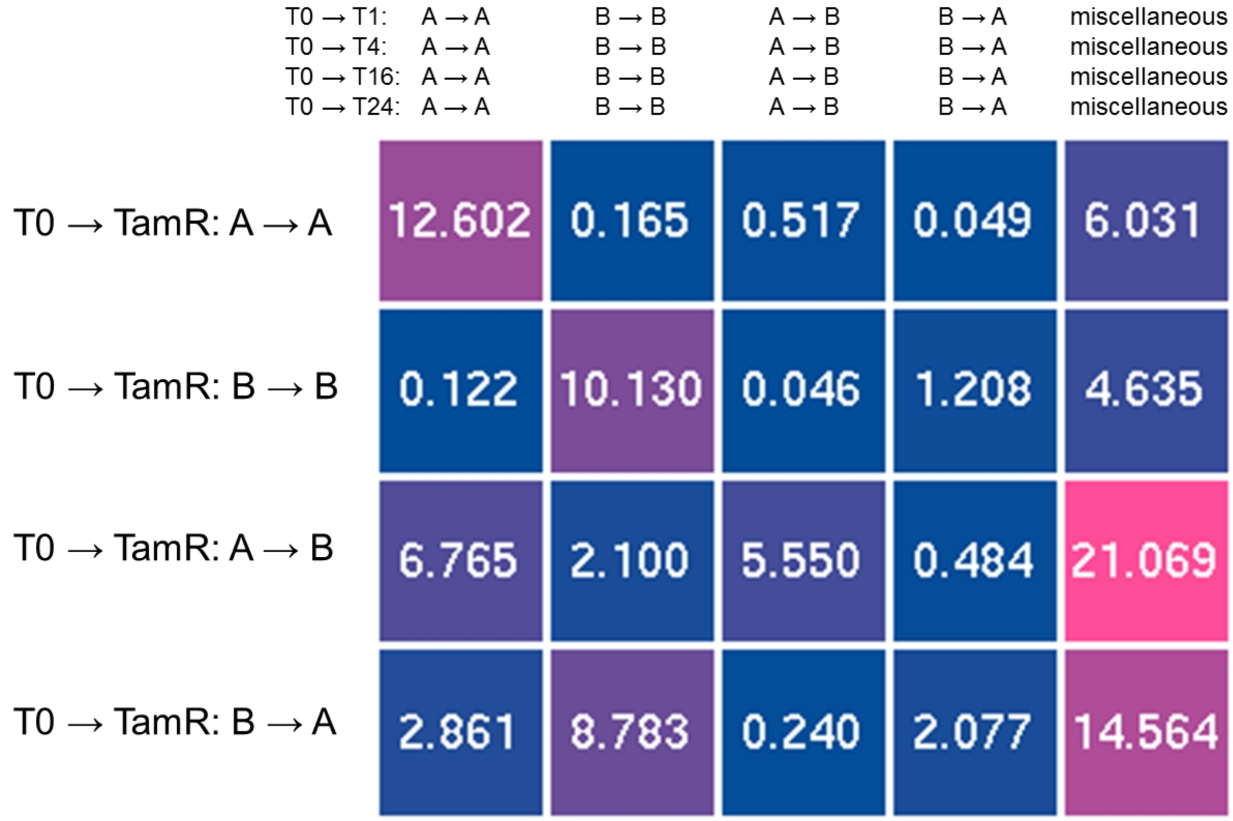

Supplementary Table 1. TCC data of E2-induced MCF7 and MCF7-TamR cells

TCC data were generated in MCF7 cells upon E2 induction for 0 hour (T0), 1 hour (T1), 4 hours (T4), 16 hours (T16), and 24 hours (T24) respetively. TCC data for MCF7-TamR cells were generated as well.

Samples	T0	T1	T4	T16	T24	TamR
Raw reads	219,147,585	226,520,745	198,742,465	211,264,913	255,965,682	190,942,974
Maped unique reads (MAPQ > 30)	145,216,469	118,566,496	108,746,461	115,179,019	166,147,762	112,299,368
Reads after filtering	81,944,028	76,502,437	76,388,172	70,200,177	97,750,738	64,992,674

Supplementary Table 2. No. of compartments in E2-induced MCF7 and MCF7-TamR cells

The compartments were produced by HiCLib python package[6]. The continuous genomic regions of positive first eigenvector were defined as compartment A (open chromatin) and the coutinuous genomic regions of negative first eigenvector was defined as compartment B (close chromatin).

Samples	Total	Compartment A	Compartment B
T0	2,067	1,038	1,029
T1	2,039	1,025	1,014
T4	2,003	1,007	996
T16	1,966	988	978
T24	1,930	970	960
TamR	2,103	1,056	1,047

Supplementary Table 3. ChIP-seq data of E2-induced MCF7 and MCF7-TamR cells

ChIP-seq data were generated in MCF7 cells upon E2 induction for 0 hour (T0), 1 hour (T1), 4 hours (T4), 16 hours (T16), and 24 hours (T24) respectively. ChIP-seq data for MCF7-TamR were generated as well.

Samples	T0	T1	T4	T16	T24	TamR
H3K4me1	79,787,909	151,392,164	66,272,350	185,013,718	61,035,053	128,994,756
H3K4me3	41,997,104	52,764,443	38,002,003	34,305,895	57,936,955	65,288,224
H3K27ac	55,162,051	46,728,735	38,300,724	33,954,483	71,532,704	114,919,065
H3K9me3	148,578,175	109,894,347	89,505,143	122,123,870	88,465,040	90,112,098
H3K27me3	160,713,062	119,366,656	82,506,113	117,436,828	69,642,505	108,119,589
CTCF	71,649,426	113,941,161	41,248,484	55,666,254	85,212,133	64,088,133
ER1	38,524,135	38,031,199	40,122,602	42,073,940	36,851,361	71,688,716
INPUT	32,060,990	33,345,392	25,143,647	20,253,802	28,416,162	59,630,935

Supplementary Table 4. RNA-seq data of MCF7 and MCF7-TamR cells

RNA-seq data for MCF7 cell line (MCF7) and MCF7-TamR cell lines were generated.

Rep: Replicate

Samples	MCF7 Rep1	MCF7 Rep2	MCF7 Rep3	TamR Rep1	TamR Rep2	TamR Rep3
Raw Reads	31,786,025	23,971,928	24,349,988	30,046,974	39,717,371	61,314,659
Mapped	30,166,810	22,737,601	23,249,967	28,978,192	38,286,180	59,290,717
Uniq	29,024,312	21,921,026	22,440,959	27,836,691	36,912,654	57,084,789

Supplementary Table 5. Primers used for 3C-qPCR

Name	Primer
TRPS1_F	AAAGACATAGGGAGATTAAAAGGATAATAC
TRPS1_R	CTTATGACAAAAATCCTATAACTCTCAACT
GALNT7_F	GATATTTATAAAGAGAAGAGGTTTAGTTGG
GALNT7_R	GTTTGAGCTCCTTATCTATTACAGTTATTA
AP4S1_F	GGAAGACTATTAATATGAATGACTGAATGT
AP4S1_R	GTATTTTATATGTCTGCTTTGTTGGAATAC
ARF6_F	CTGGTAGTTTCTCTCAAGTAGTGAGTTAAG
ARF6_R	ACATTTTAGATCTTAGTAGAACTCCATTAC
BAZ1A_F	ATAGGTATGTATAACTCCCTGATAACAATA
BAZ1A_R	AATACTCAAATGAATAGACTACCTAAACTG
SPTSSA_F	GAATGTAATGTTTAAGCTGTAGAAAGTATG
SPTSSA_R	ACTGTCATAGTTCTATGTTTATTAGTGATG
DLGAP5_F	ATATTATATCCTAAATCGACATCTTAGCAC
DLGAP5_R	ATCCCTTATGTTAACTAGATGATAGATTCA

Supplementary References:

1. Lun ATL, Smyth GK. diffHic: a Bioconductor package to detect differential genomic interactions in Hi-C data. *BMC Bioinformatics*, **16**, 258 (2015).

2. Stark R, Brown G. DiffBind: differential binding analysis of ChIP-Seq peak data. http://bioconductor.org/packages/release/bioc/vignettes/DiffBind/inst/doc/DiffBind.pdf. (2011).

3. Ross-Innes CS, Stark R, Teschendorff AE, Holmes KA, Ali HR, Dunning MJ, Brown GD, Gojis O, Ellis IO, Green AR, Ali S, Chin S, Palmieri C, Caldas C, Carroll JS. Differential oestrogen receptor binding is associated with clinical outcome in breast cancer. *Nature* 481, -4 (2012).

4. Subramanian A, Tamayo P, Mootha VK, Mukherjee S, Ebert BL, Gillette MA, Paulovich A, Pomeroy SL, Golub TR, Lander ES, Mesirov JP. Gene set enrichment analysis: a knowledge-based approach for interpreting genome-wide expression profiles. *Proc Natl Acad Sci U S A*. Oct 25;102(43):15545-50 (2005).

5. Lanczky A, Nagy A, Bottai G, Munkacsy G, Paladini L, Szabo A, Santarpia L, Gyorffy B. miRpower: a web-tool to validate survival-associated miRNAs utilizing expression data from 2,178 breast cancer patients. *Breast Cancer Res Treat*. 160(3):439-446 (2016).

6. Imakaev, M., Fudenberg, G., McCord, R.P., Naumova, N., Goloborodko, A., Lajoie, B.R., Dekker, J., and Mirny, L.A. Iterative correction of Hi-C data reveals hallmarks of chromosome organization. *Nat. Methods* 9, 999–1003 (2012).
