# Supplementary material for: Temporal dynamic reorganization of 3D chromatin architecture in hormone-induced breast cancer and endocrine resistance
Source: Nat Commun. 2019 Apr 3;10:1522. doi: 10.1038/s41467-019-09320-9 (PMC6447566; doi:10.1038/s41467-019-09320-9)
Supplement: Supplementary file 3 — Description of Additional Supplementary Files [file 41467_2019_9320_MOESM3_ESM.pdf]

Description of Additional Supplementary Information

File Name: Supplementary Data 1
Description: Identified compartments of TA-HCC, TA-ETC, TA-LTC, TA-LDC, TA-MDC and TA-HDC

File Name: Supplementary Data 2
Description: Dynamically differential expressed genes (DDEGs) and their expression value (log2) in MDC and HDC

File Name: Supplementary Data 3
Description: Dynamically differential expressed genes (DDEGs) and their expression value (log2) in TA-MDC&TA-HDC

File Name: Supplementary Data 4
Description: HOMER loops associated with TDEGs in theTA-MDC and TA-HDC

File Name: Supplementary Data 5
Description: TamR-specific differentially expressed genes (TDEGs) in ERα-related promoter-enhancer HOMER loops located at TA-MDC&TA-HDC
